# System Model Network for Adipose Tissue Signatures Related to Weight Changes in Response to Calorie Restriction and Subsequent Weight Maintenance

**DOI:** 10.1371/journal.pcbi.1004047

**Published:** 2015-01-15

**Authors:** Emilie Montastier, Nathalie Villa-Vialaneix, Sylvie Caspar-Bauguil, Petr Hlavaty, Eva Tvrzicka, Ignacio Gonzalez, Wim H. M. Saris, Dominique Langin, Marie Kunesova, Nathalie Viguerie

**Affiliations:** 1 Institut National de la Santé et de la Recherche Médicale (INSERM), UMR1048, Obesity Research Laboratory, Institute of Metabolic and Cardiovascular Diseases (I2MC), Toulouse, France; 2 University of Toulouse, UMR1048, Paul Sabatier University, Toulouse, France; 3 Toulouse University Hospitals, Departments of Clinical Biochemistry and Nutrition, Toulouse, France; 4 INRA, UR875 MIAT, Castanet Tolosan, France; 5 Statistique, Analyse, Modélisation Multidisciplinaire (SAMM), Université Paris 1, Paris, France; 6 Institute of Endocrinology, Obesity Management Centre, Prague, Czech Republic; 7 Fourth Department of Internal Medicine, 1st Medical School, Charles University, Prague, Czech Republic; 8 Department of Human Biology, NUTRIM School for Nutrition, Toxicology and Metabolism, Maastricht University Medical Centre, Maastricht, The Netherlands; Mount Sinai School of Medicine, UNITED STATES

## Abstract

Nutrigenomics investigates relationships between nutrients and all genome-encoded molecular entities. This holistic approach requires systems biology to scrutinize the effects of diet on tissue biology. To decipher the adipose tissue (AT) response to diet induced weight changes we focused on key molecular (lipids and transcripts) AT species during a longitudinal dietary intervention. To obtain a systems model, a network approach was used to combine all sets of variables (bio-clinical, fatty acids and mRNA levels) and get an overview of their interactions. AT fatty acids and mRNA levels were quantified in 135 obese women at baseline, after an 8-week low calorie diet (LCD) and after 6 months of ad libitum weight maintenance diet (WMD). After LCD, individuals were stratified a posteriori according to weight change during WMD. A 3 steps approach was used to infer a global model involving the 3 sets of variables. It consisted in inferring intra-omic networks with sparse partial correlations and inter-omic networks with regularized canonical correlation analysis and finally combining the obtained omic-specific network in a single global model. The resulting networks were analyzed using node clustering, systematic important node extraction and cluster comparisons. Overall, AT showed both constant and phase-specific biological signatures in response to dietary intervention. AT from women regaining weight displayed growth factors, angiogenesis and proliferation signaling signatures, suggesting unfavorable tissue hyperplasia. By contrast, after LCD a strong positive relationship between AT myristoleic acid (a fatty acid with low AT level) content and de novo lipogenesis mRNAs was found. This relationship was also observed, after WMD, in the group of women that continued to lose weight. This original system biology approach provides novel insight in the AT response to weight control by highlighting the central role of myristoleic acid that may account for the beneficial effects of weight loss.

## Author Summary

Obesity is an excess fat mass leading to metabolic diseases. Dietary management is a conventional strategy to promote weight loss. As energy buffering, in the form of esterified fatty acids, and secretory organ, the adipose tissue has a pivotal role in obesity and its related complications. A comprehensive insight of adipose tissue response during and after calorie restriction might improve obesity management. Modern nutrition research study the impact of diet on health by combining multiple datasets to provide an holistic view of tissue physiopathology. To identify significant clusters of fatty acids, transcripts or bio-clinical parameters related to weight change along calorie restriction and subsequent weight follow-up in obese individuals, the issue of different datasets integration must be resolved. Here, we implemented an innovative multistep approach to infer multi-data networks and compare clusters of network components. This original strategy highlighted an unexpected central role of a minor adipose tissue fatty acid, myristoleic acid, which is not provided by food. Its link to transcripts encoding enzymes from a pathway converting glucose into fat that mediates favorable metabolic effects makes myristoleic acid a key factor of the positive impact of fat mass reduction.

## Introduction

The main function of adipose tissue (AT) is to store excess energy as triglycerides and to release non-esterified fatty acids (FAs) for other tissues during periods of energy demand. AT also releases numerous peptidic/proteic and lipidic factors with signaling functions [1–3]. Obesity is characterized by an excess fat mass with deleterious health consequences. AT expansion results in dysfunctional non-esterified FA release and imbalance in production of anti/pro-inflammatory mediators [4]. Most of the obesity-related metabolic disturbances are reversible with weight loss [5]. However in obese individuals, weight fluctuations are frequent since individuals involved in dieting-induced weight loss are often unsuccessful at long last [6, 7]. Adaptations occurring in AT during dietary weight management programs remain unclear especially regarding weight control after dieting [8]. The FA composition of AT reflects balance between exogenous FAs from food, triglyceride hydrolysis/synthesis and FA synthesis from glucose-derived acetylCoA, so-called *de novo* lipogenesis (DNL). Studies on FA composition of AT during weight control trials are scarce [9, 10]. Low 16:1(cis-9) (palmitoleic acid) and 14:1(cis-9) (myristoleic acid) may predict favorable weight control outcome [11].

Omics, especially transcriptome studies, have proved great potential in clarifying the role of AT biology with respect to response in weight controlling trials [12]. However, analyses based on single omics often do not provide enough information to understand biology. The integration of multiple omics may give a better understanding of a biological system as a whole. Global network-based approaches authorize multiple datasets analyses and carry the advantage of highlighting functionally related pathways and biological entities of potential relevance as hubs [13]. Networks are valuable models to dissect complex traits [14]. However, integrative analysis of datasets of different data types raises the issue of different scales of the multiple datasets. In gene expression networks, clusters are more robust than individual interactions [15]. Multivariate statistical approaches were recently developed to jointly analyze omics datasets, dealing with high dimension and using variable selection [16].

The present study aimed at revealing the characteristics of AT biological networks relevant to clinical traits during a long-term dietary intervention (DI) including calorie restriction and *ad libitum* follow-up after weight loss. Studies on human AT gene expression or lipidomic profiles from a systems biology point of view have only been reported at baseline [17, 18] but not during DI. Network modeling has recently been applied using metagenomic, plasma and AT inflammatory markers to predict weight changes during stabilized weight loss [19]. To our knowledge, no study has jointly investigated AT lipidic and gene expression profiles, especially during long-term DIs.

Here, the global AT networks were computed using FAs, mRNA levels, clinical risk factors and biochemical markers according to weight changes in the same individuals. Our purpose was to identify common as well as differential signatures with relationship to bio-clinical factors. The identification of novel AT features associated with weight regulation may influence our understanding of weight control and authorize new advances in obesity management.

## Materials and Methods

### Source data

**Ethics statement**. The samples investigated in this paper were collected from 2006 to 2007 during the DiOGenes study, a pan-European randomized DI trial which was approved by the ethics committees of each of the 8 European centres participating to the program (registration no. NCT00390637). Written informed consent was obtained from each patient according to the local ethics committee of the participating countries: 1, Medical Ethics Committee of the University Hospital Maastricht and Maastricht University, The Netherlands; 2, The Committees on Biomedical Research Ethics for the Capital region of Denmark, Denmark; 3, Suffolk Local Research Ethics Committee, UK; 4, University of Crete Ethics Committee, Greece; 5, the Ethics Commission of the University of Potsdam; 6, Research Ethics Committee at the University of Navarra, Spain; 7, Ethical Committee of the Institute of Endocrinology, Czech Republic; 8, Ethical Committee to the National Transport Multiprofile Hospital in Sofia, Bulgaria.

**Figure 1:**
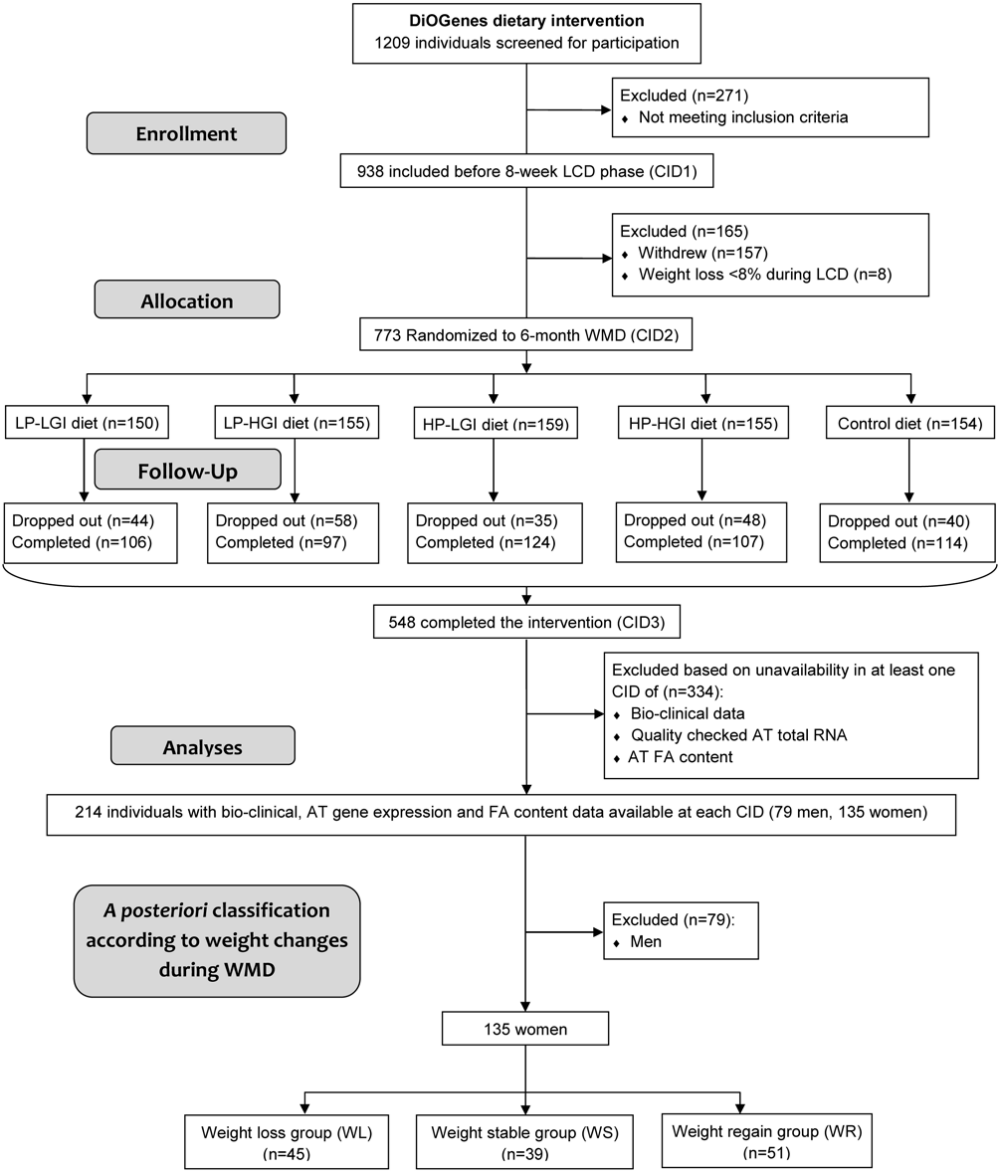
Flowchart for individuals’ selection from the DiOGenes cohort. Participants entering subsequent phases of the study as well as dropouts are indicated in total. AT, adipose tissue; CID, clinical investigation day; FA, fatty acids; HGI, high glycemic index; HP, high protein content; LCD, low calorie diet; LGI, low glycemic index; LP, low protein content; WMD, weight maintenance diet.

**Study design**. The data presented in this paper are part of those collected during the DiOGenes study (contact information at www.diogenes-eu.org) The DiOGenes project investigated the effects of diets with different content of protein and glycemic index on weight-loss maintenance and metabolic and cardiovascular risk factors after a phase of calorie restriction, in obese/overweight individuals. The trial protocol and supporting CONSORT checklist are available as supporting information; see S1 Protocol and S1 Checklist. Healthy overweight (body mass index (BMI) ≥27 kg/m2) individuals, aged <65 years were eligible for the study. Exclusion criteria were BMI 45 kg/m2, liver or kidney diseases, cardiovascular diseases, diabetes mellitus (type 1 or type 2), special diets/eating disorders, systemic infections/chronic diseases, cancer within the last 10 years, weight change >3 kg within the previous 3 months, and other clinical disorders or use of prescription medication that might interfere with the outcome of the study. A detailed description of inclusion and exclusion criteria has been published previously [20]. BMI was calculated by dividing weight in kilograms by the square of height in meters. Waist circumference was measured between the bottom of the ribs and the top of the hip bone. A detailed description of the DiOGenes intervention trial and main outcomes can be found in previous core publications [20–22]. Briefly, after the first clinical investigation day (baseline), eligible individuals followed an active weight loss phase of 8-week low calorie (3.3–4.2 MJ/d) diet (LCD) using commercial meal replacements (Modifast, Nutrition et Santé). The individuals with ≥ 8% of initial body weight loss during LCD were randomized into one of five *ad libitum* weight maintenance diets (WMD) for 6 months: 4 diets combining high and low protein content with high and low glycemic index of carbohydrates, and a control low fat (25–30% energy) diet according to National dietary guidelines on healthy diets [22]. During WMD, the individuals were provided dietary instruction as described in [22]. Dietary intake was assessed at screening, 4 weeks after the beginning and at the end of WMD. The subjects were asked to complete a 3-day weighed food record, including 2-week days and 1 weekend day. Dietary records were validated by a nutritionist. Clinical investigations including anthropometric measures (height, weight, waist circumference, body composition), blood pressure measurements, fasting blood sampling, and subcutaneous AT biopsies were performed at baseline (BAS) and at the end of each phase. All procedures were standardized between the 8 study centers across Europe [21]. Fig. 1 displays the organizational flowchart through the trial protocol and the individuals’ selection from the DiOGenes cohort for the present study.

**Patients and adipose tissue study**. Biopsy samples were stored at -80°C until total RNA and FA extractions. The lipid fraction was extracted from the fat cake produced during total RNA extraction using gas chromatography as described in [11]. The list of FA extracted from the lipid fraction is presented in S1 Table. After RNA extraction the mRNA levels of a panel of 221 genes selected from previous published and unpublished DNA microarray analyses on limited number of individuals as described in [23] was assessed using high throughput real-time PCR as described in [24]. S2 Table describes these genes according to biological pathways and the biological function of the protein encoded. The list includes 68 genes previously shown as markers of subcutaneous AT from obese insulin resistant subjects with metabolic syndrome [25], 65 genes described as markers of subcutaneous AT from lean individuals [25], 33 genes selected from previous caloric restriction induced weight loss studies [26, 27], 27 markers of weight changes after caloric restriction [28], and 28 unpublished predictors of weight change to distinguish between those subjects that will regain weight after LCD from those that will succeed weight maintaining based on the AT transcriptome at baseline or after the caloric restriction phase. These genes encoded proteins involved in various pathways such as metabolism (47.5% of the transcripts), immune response (19.5%), transport (4.5%), cell and tissue structure (3.6%), signal transduction (2.3%) and response to stress (1.4%). A subgroup of the DiOGenes cohort was selected based on the availability of the FA and gene profiling quality data. Here, among the 214 individuals with both AT gene expression and FA content available at all steps of the DI, *i*.*e*. BAS, LCD and WMD, only premenopausal women were studied (n = 135). After LCD, the women were classified *a posteriori* into 3 separate groups according to weight changes during WMD, calculated by subtracting body weight at LCD to body weight at WMD. Subjects who experienced a weight loss or a weight regain greater or equal to 2 kg during WMD were classified as weight losers (WL) (n = 45) or weight regainers (WR) (n = 51), respectively. Individuals with weight change of less than 2 kg were classified as stable weight (WS, n = 39).

**Data availability statement**. Raw and processed RT-qPCR data files were deposited at the Gene Expression Omnibus depository and are available under series accession number GSE60946. Other data data are available upon request.

### Statistical analysis of clinical data, mRNA levels and adipose tissue fatty acids

Data were first analyzed by multivariate statistical methods using principal component analysis to detect center or diet group biases and mean-centered transformed if needed. Gaussian distribution of data was tested using the Kolmogorov–Smirnov test and log transformed adequately. Differences in clinical data, mRNA and FAs between BAS, end of LCD and end of WMD were tested using one-factor repeated measure ANOVA with Bonferroni post-hoc test. The differences between each group (WL, WR and WS) at each step of the DI were tested with one-factor ANOVA and Bonferroni post-hoc test. Fatty acids and gene expression data were controlled for multiple testing by using Benjamini-Hochberg P value correction (q-value) [29]. Analyses were performed with SPSS Statistics 17.0 software (SPSS Inc., Chicago, Ill).

### Network inference

**Figure 2:**
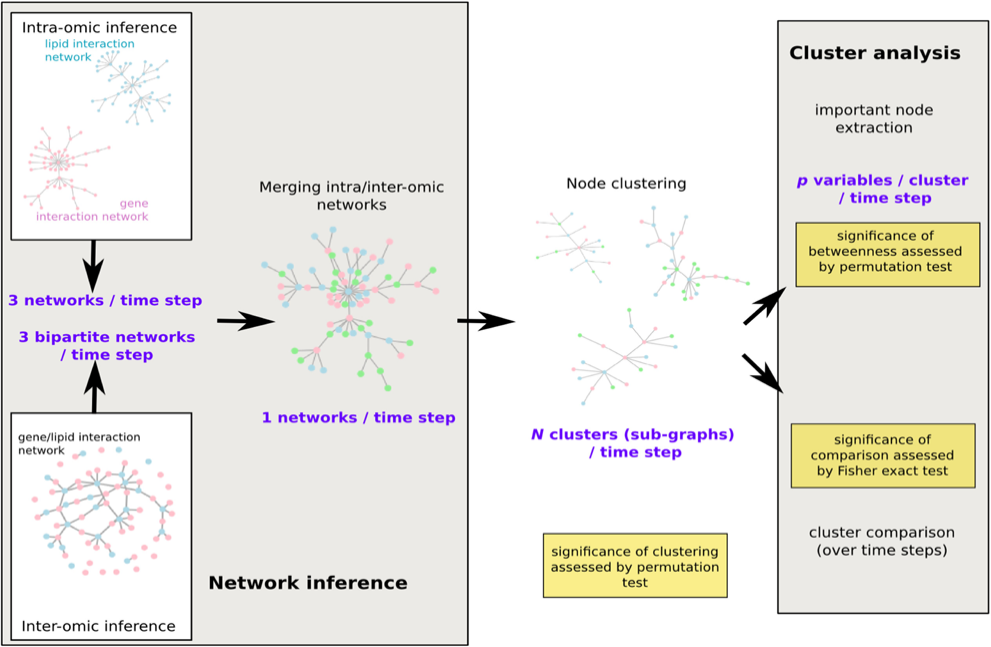
Workflow of the network analysis. Intra- and inter-omics networks were first inferred separately before a global merging for each time step: at baseline, after a 8-week low calorie diet and after weight maintenance diet (3 groups). Node clustering was then performed and clusters were systematically analyzed with most important nodes extraction and across time comparison. Resulting products of each step are given in purple and significant tests to assess the validity of the approach are given in yellow.

The network analysis was performed as illustrated in Fig. 2: for BAS, the end of LCD and the 3 groups at the end of WMD (WR, WL and WS), a system model was designed using a global network. The network was built using a 3 step approach. A first step consisted in inferring a network in each set of variables (bio-clinical, FAs and mRNA level) using a sparse Graphical Gaussian Model (GGM, [30]). This model is based on the assumption that, in each set of variables, the distribution of the variables, (*X^j^*)_*j = 1* … *p’*_ is Gaussian N(0, ∑) and that the observations obtained for all individuals are independent and identically distributed. The method then unravels the conditional dependency structure of the variables, *i*.*e*., defines a network whose edges correspond to positive or negative partial correlations P(*X^j^, X^j’^*|(*X^k^*)_*k≠j, j’*_) Using a maximum likelihood approach, the method performs an edge selection, simultaneously to the estimation of partial correlations. Unlike simple correlation, partial correlation is a mean to assess direct correlations between pairs of variables, independently of the other variables and is thus closer to a causality relation than simple correlation. The number of selected edges was chosen according to the description given in step 3 below. A second step consisted in inferring a network between each pairs of two different sets of variables among bio-clinical, FAs and mRNA level sets. To do so, we used the approach that was proven successful to infer a gene/phenotype network in [31]: regularization canonical correlation analysis (CCA, [16, 32]). The additional regularization constraint was used to deal with the large number of variables as compared to the number of observations. The number of selected edges was chosen according to the description given in step 3 below. A third step consisted in merging the 3 networks obtained in the first step with the 3 networks obtained in the second step. As the number of variables in the 3 datasets was very different (from 15 bio-clinical variables up to 221 gene expressions), a naive strategy consisting in estimating the selected edges in each set of variables (or in each pair of two sets) in a same manner would have led to give too much importance on the largest set of variables, *i*.*e*., to the gene expression dataset. The number of selected edges was thus adjusted to be equal to the number of nodes in each set (or pair of sets) of variables, leading to smaller densities for the largest networks.

The first step of the analysis was performed using the R package glasso (cran.r-project.org/web/packages/glasso) and the second step using the R package mixOmics (http://perso.math.univ-toulouse.fr/mixomics).

**Global network analysis**. To stress out the macro-structure of the network, a spin-glass model and simulated annealing were used to maximize the modularity quality measure [33] and obtain a vertex clustering [34] for all 5 networks.

The significance of the clustering was assessed using a permutation test as described in [35]: the clustering was declared significant if the obtained modularity was larger than the maximum modularity found over 100 random graphs with the same degree distribution than the graph under study. Random graphs with identical degree distributions were generated using a permutation of the edges as justified by [36].

**Sub-network analysis**. Significance of the betweenness within a cluster was assessed using a permutation test to check if the betweenness was significantly high regarding the node’s degree in its cluster. A significant result (p<0.05) indicates a node more central than expected in the graph and a non-significant (p≥0.05) result indicates a node which centrality is expected for the node’s degree. For nodes with a high degree (so-called hubs), a non-significant result does not however indicate that the node is not important within its cluster: its importance is already acknowledged by its many connections with the other nodes. But, provided its degree, it is not particularly central. Significant betweenness was thus used as a measure of importance of the hubs in the networks (even though hubs were systematically investigated, it provided an additional information on the node’s critical role). The permutation test was performed in a way similar to the modularity test: the highest betweenness over 100 random graphs with the same degree distribution was compared to all observed betweenness. The nodes with an observed betweenness in the top 5% were declared significant. The network analysis (node clustering and betweenness calculation) was performed with the R package igraph (igraph.org; [37]).

Finally, clusters with identical central nodes in two different networks were tested for the significance of the number of common nodes using a Fisher exact test with the set of all variables as reference: pairs of clusters with a p-value smaller than 5% in the Fisher exact test are those that have a larger number of common nodes than what was expected by random chance only.

Sub-graphs (clusters) were laid out using force-based algorithms in Gephi 0.8.2 software (gephi.org, [38, 39]). Nodes’ sizes indicate degree, *i*.*e*., the number of edges adjacent to the node. Nodes with the largest degrees, called hubs, were systematically extracted. Nodes’ colors and font size indicate betweenness centrality, a measure that counts how often a node appears on shortest paths between two other nodes in the network. Therefore, betweenness centrality indicates nodes that are the most likely to disconnect the network if removed. The variables are connected by an edge only if they have been selected by the sparse estimation. Edge thickness is proportional to the strength of the correlation (CCA) or of the partial correlation (GGM) but should only be compared for a given set of estimation (*i*.*e*., partial correlation strength between two pairs of genes can be compared but should not be compared to correlation between a gene and a FA or a bio-clinical parameter).

The biological functions represented by mRNAs from each cluster were searched using Ingenuity Pathways Analysis (IPA) software version 7.5 (Ingenuity Systems, Redwood City, CA). The significance of canonical pathways was tested using the Fisher Exact test with the set of 221 genes as reference. Data were controlled for multiple testing by using Benjamini-Hochberg P value correction.

## Results

### Obese individuals’ description

Baseline anthropometric and clinical characteristics of the 135 women are displayed in Table 1. After the end of LCD, individuals were *a posteriori* classified into 3 groups according to weight changes during WMD. To ensure that there was no striking between group difference at baseline and after LCD, bio-clinical variables, gene expression and FA profiles were also analyzed *a posteriori* according to weight control classification.

**Table 1:** Baseline anthropometric and clinical characteristics of the 135 obese women.

**Parameters**	**Mean ± SEM**	**Range**	**Median**
Age (y)	42.4 ± 0.6	24–58	42.0
Dietary intake (kJ/day)	8568 ± 225	2116–15677	8557
Weight (kg)	94.0 ± 1.3	66.6–149.7	91.6
BMI (kg/m²)	34.3 ± 0.4	26.8–47.7	33.4
Fat Mass (%)	43.4 ± 0.5	28.6–56.6	42.7
Waist (cm)	103 ± 1	74–142	102
SBP (mmHg)	124 ± 1	89–165	122
DBP (mmHg)	77 ± 1	50–104	78
Total cholesterol (mmol/l)	4.8 ± 0.1	2.3–6.9	4.8
Triglycerides (mmol/l)	1.3 ± 0.05	0.4–3.1	1.1
HDL cholesterol (mmol/l)	1.3 ± 0.03	0.6–2.2	1.3
LDL cholesterol (mmol/l)	3.0 ± 0.1	1.3–5.0	2.9
Fasting glucose (mmol/l)	5.0 ± 0.1	1.7–7.6	5.0
Fasting insulin (µIU/ml)	9.8 ± 0.5	2.5–31.8	8.4
HOMA-IR	2.2 ± 0.1	0.4–10.3	1.8
Fructosamine (µmol/l)	207.1 ± 2.2	127–338	208
Adiponectin (µg/ml)	10.7 ± 0.5	0.6–30.1	9.8
CRP (mg/l)	4.2 ± 0.3	0.3–21.9	3.3

BMI, body mass index; CRP, C reactive protein; DBP, diastolic blood pressure; HDL, high density lipoprotein; HOMA-IR, homeostatic model assessment of insulin resistance; LDL, low density lipoprotein; SBP, systolic blood pressure.

At baseline, women from WL group had higher weight and BMI than those from WS groups (Table 2). Weight loss induced by LCD was similar in the 3 groups even though mean weight in WL group remained higher than in WS group after the LCD. Plasma adiponectin was higher at baseline in WL group compared to WR and WS groups. During LCD, there was no intergroup difference in bio-clinical changes. All parameters improved except plasma fructosamine and adiponectin. S3 Table displays the anthropometric and clinical characteristics at the end of the weight maintenance phase according to weight control group and by randomization arm. During WMD, women from WL group lost 7.0 ± 0.4 kg compared to the end of LCD and those from WR group regained 5.0 ± 0.4 kg. Adiponectin improved during WMD only in WL group. There was no difference regarding age, center (data not shown) or distribution of the 5 WMD dietary arms between groups (S3 Table). There was no intergroup difference in changes in dietary intake along DI (S1 Fig.).

**Table 2:** Anthropometric and clinical characteristics of women according to weight control groups during dietary intervention.

	**WR (n = 51)**			**WS (n = 39)**			**WL (n = 45)**		
**Age at screening (y)**	**42.4 ± 0.9**			**41.5 ± 1.1**			**43.2 ± 1.0**		
	**BAS**	**LCD**	**WMD**	**BAS**	**LCD**	**WMD**	**BAS**	**LCD**	**WMD**
Weight (kg)	92.5 ± 2.5	82.7 ± 1.9^*1*^	87.7 ± 2.0^*1*, 3^	90.7 ± 1.9^*3*^	80.4 ± 1.5^*1, 3*^	80.5 ± 1.5^*1, 4*^	99.8 ± 3.7	88.0 ± 2.5^*1*^	81.2 ± 2.3^*1, 2*^
BMI (kg/m²)	33.8 ± 0.7	30.3 ± 0.6^*1*^	32.2 ± 0.6^*1, 2, 3*^	33.3 ± 0.6^*3*^	29.6 ± 0.5^*1*^	29.7 ± 0.6^*1, 4*^	35.9 ± 0.8	31.8 ± 0.7^*1*^	19.3 ± 0.6^*1, 2*^
Fat Mass (%)	43.9 ± 0.7	40.2 ± 1.1^*1*^	41.5 ± 0.9^*1*^	41.9 ± 0.8	38.4 ± 1.0^*1*^	37.5 ± 0.8^*1, 4*^	44.2 ± 0.9	39.6 ± 0.9^*1*^	36.3 ± 0.9^*1, 4*^
Waist (cm)	103 ± 1	95 ± 1^*1*^	98 ± 1^*1, 3*^	101 ± 1	91 ± 1^*1*^	92 ± 1^*1, 4*^	105 ± 2	95 ± 2^*1*^	90 ± 2^*1, 2*^
SBP (mmHg)	121 ± 2	113 ± 2^*1*^	119 ± 1	123 ± 2	117 ± 2^*1*^	120 ± 2	128 ± 2	120 ± 2^*1*^	121 ± 2^*1*^
DBP (mmHg)	74 ± 1^*4*^	71 ± 1	74 ± 2	77 ± 2	73 ± 2	74 ± 2	80 ± 2	75 ± 1^*1*^	75 ± 1^*1*^
Total Cholesterol (mmol/l)	4.8 ± 0.1	4.3 ± 0.1^*1*^	4.9 ± 0.1^*2*^	4.9 ± 0.1	4.2 ± 0.1^*1*^	4.8 ± 0.1^*2*^	4.8 ± 0.1	4.3 ± 0.1^*1*^	4.8 ± 0.1^*2*^
Triglycerides (mmol/l)	1.3 ± 0.08	1.1 ± 0.06	1.2 ± 0.07	1.2 ± 0.08	0.9 ± 0.06^*1*^	0.9 ± 0.05^*1, 5*^	1.3 ± 0.09	1.2 ± 0.07	1.1 ± 0.07^*1*^
HDL (mmol/l)	1.3 ± 0.04	1.2 ± 0.04^*1*^	1.44± 0.04^*2*^	1.3 ± 0.06	1.2 ± 0.06	1.5 ± 0.07^*2*^	1.3 ± 0.06	1.2 ± 0.04^*1*^	1.5 ± 0.05^*1, 2*^
LDL (mmol/l)	2.9 ± 0.1	2.6 ± 0.1	2.9 ± 0.1^*2*^	3.0 ± 0.1	2.5 ± 0.1^*1*^	2.9± 0.1^*2*^	2.9 ± 0.1	2.6 ± 0.1	2.8 ± 0.1^*2*^
Fasting glucose (mmol/l)	5.1 ± 0.1	4.7 ± 0.1^*1*^	4.9 ± 0.1^*2*^	5.1 ± 0.1	4.8 ± 0.1	4.9 ± 0.1	5.0 ± 0.1	4.8 ± 0.1	4.8 ± 0.1
Fasting insulin (µIU/ml)	9.6 ± 0.8	7.2 ± 0.5^*1*^	8.6 ± 0.6	10.3 ± 1.0	6.5 ± 0.6^*1*^	6.8 ± 0.6^*1*^	9.7 ± 0.9	8.0 ± 0.81	8.4 ± 1.0
HOMA-IR	2.2 ± 0.2	1.5 ± 0.1^*1*^	1.8 ± 0.1	2.3 ± 0.3	1.3 ± 0.1^*1*^	1.5 ± 0.1^*1*^	2.2 ± 0.3	1.6 ± 0.2	1.8 ± 0.3
Fructosamine (µmol/l)	207 ± 3	207 ± 3	215 ± 3^*2*^	209 ± 50	209 ± 4	219 ± 4^*1, 2*^	205 ± 4	211 ± 3	218 ± 3
Adiponectin (µg/ml)	10.2 ± 0.6	10.7 ± 0.5	11.6 ± 0.6	9.1 ± 0.6^*3*^	9.9 ± 0.6	10.7 ± 0.8^*3*^	12.9 ± 1.0^*4*^	11.2 ± 0.6	13.6 ± 0.8^*2*^
CRP (mg/l)	4.0 ± 0.5	2.5 ± 0.3^*1*^	2.9 ± 0.4	4.4 ± 0.6	3.6 ± 0.6	2.1 ± 0.4^*1*^	4.3 ± 0.6	4.2 ± 0.6	3.4 ± 0.5

Anthropometric, clinical and plasma parameters were determined at baseline (BAS), after 8 weeks of low calorie diet (LCD), and after 6 months of weight maintenance diet (WMD) according to weight control groups during dietary intervention (WR, weight regain group; WS, weight stable group; WL, weight loss group). BMI, body mass index; CRP, C reactive protein; DBP, diastolic blood pressure; HDL, high density lipoprotein; HOMA-IR, homeostatic model assessment of insulin resistance; LDL, low density lipoprotein; SBP, systolic blood pressure. Variables are shown as means ± SEM. The effect of time was analyzed by repeated measures ANOVA with Bonferroni post hoc test: ^*1*^ p < 0.05, data significantly different from BAS. ^*2*^ p < 0.05, data significantly different from LCD. Between group difference was analyzed by one-way ANOVA with Bonferroni post hoc test: ^*3*^ p < 0.05, data significantly different from WL group. ^*4*^ p < 0.05, data significantly different from WR group.

### Adipose tissue gene expression and fatty acid content

A bunch of 221 mRNA (S2 Table) selected from previous AT investigations using microarrays was quantitatively assessed using RT-qPCR. Among these genes, 155 genes were down-regulated during LCD. The most representative pattern was a down-regulation during LCD and up-regulation during WMD. The most regulated genes in the 3 groups, *SCD* and *FASN*, encoded enzymes for different steps of FA synthesis, stearoyl CoA desaturase and fatty acid synthase, respectively (S2 Fig.).

S3 Table displays the AT changes in FA composition. At baseline, in WL group, AT had higher percentages of polyunsaturated FAs (PUFAs) and lower saturated FAs (SFAs) and mono unsaturated FAs (MUFAs) compared with other groups. SFAs and MUFAs exhibited the most representative changing course during DI. During LCD, in WL group, 2 SFAs (12:0 and 14:0) and 2 MUFAs (14:1(cis-9) and 16:1(cis-9)) AT content decreased. Three other MUFAs (18:1(cis-9), 20:1(cis-11), 16:1(cis-7)), and 4 PUFAs, including 20:4(cis-5, 8, 11, 14), increased. Altogether, after WMD, the AT from WL and WS groups had similar FA profile than after LCD. In the WR group, the FA content returned to baseline values. The greatest changes were an increase in 12:0, and 14:1(cis-9) and a decrease of 18:1(cis-9) percentages.

S3 Fig. displays SCD activities assessed using 14:1(cis-9)/14:0, 16:1(cis-9)/16:0 and 18:1(cis-9)/18:0 ratios and showed no between group difference at baseline and after LCD. At the end of WMD, 14:1(cis-9)/14:0 and 16:1(cis-9)/16:0, but not 18:1(cis-9)/18:0, were higher in WR compared to WL group.

### Adipose tissue network inference and clustering

Network inference was performed using the 3 step inference method (see Materials and Methods) at baseline, at the end of LCD, and in the 3 groups at the end of WMD, resulting in 5 global networks. Then, to stress out the macro structure of the network, a vertex clustering was performed.

All 5 clustering performed on the 5 global networks were found to have a significantly high modularity, proving the relevance of the sub-graphs (clusters).

**Figure 3:**
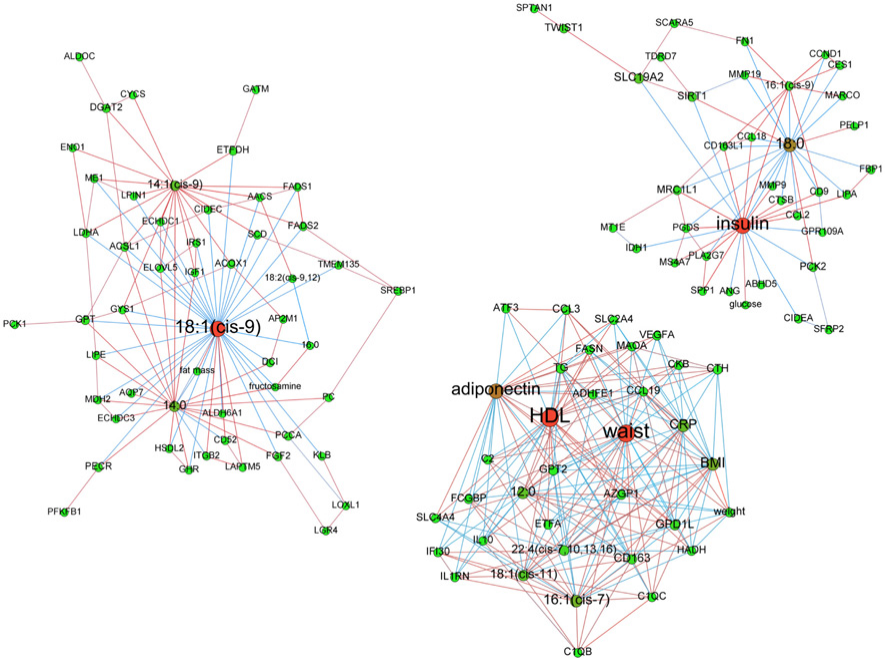
Baseline adipose tissue networks of obese women. A sparse Graphical Gaussian Model (GGM) was used to estimate partial correlations in each set of variables and regularized canonical correlation analysis (CCA) was used to assess links between paired sets of variable. Clustering was performed using a spin glass model and simulated annealing. This analysis displays the variables that are connected independently from other variables. Graphs were laid out using force-based algorithms in Gephi 0.8.2 software. Nodes’ colors and font size indicate betweenness centrality. The red nodes have the highest betweenness and the green nodes the lowest one. Edge thickness is proportional to the strength of the correlation (CCA) or of the partial correlation (GGM). Edge color indicates the correlation sign: red for positive correlations and blue for negative ones. BMI, body mass index; CRP, C reactive protein; DBP, diastolic blood pressure; HDL, high density lipoprotein; LDL, low density lipoprotein; SBP, systolic blood pressure; TG, plasma triglycerides; waist, waist circumference.

**At baseline**. Before LCD, among 14 clusters detected, 9 displayed more than 6 vertices. Insulin, waist circumference and 18:1(cis-9) were central nodes of 3 of the clusters containing at least 2 types or variables (bio-clinical, FAs or mRNAs) (Fig. 3).

Insulin was the variable with most significant betweenness centrality (p-value = 0.03) among the most central nodes of the 3 clusters. The insulin-centered cluster contained plasma glucose and mRNA encoding proteins involved in “Adhesion and Diapedesis” as major canonical pathway according to IPA analysis. This included various cytokines (*CCL2, CCL18*) and metalloproteases (*MMP9, MMP19)* with positive correlation to fasting insulin. Most of these genes were negatively linked to 18:0 and positively linked to 16:1(cis-9). The module whose hubs were waist circumference (degree: 27; p-value of waist circumference betweenness centrality = 0.39—not significant) and HDL (betweenness centrality p-value = 0.36—not significant) showed respectively positive and negative correlations with genes involved in an “Immune Response” gene expression IPA signature (*CD163*, *CCL3*, *CCL19*, *C1QC*, *C2*, *IL10* and *FCGBP*). Adiponectin was negatively connected to part of these immune response genes and 18:1(cis-11). Among genes negatively connected to waist circumference were *AZGP1* and *GPD1L*, whose lower expression in AT from metabolic syndrome (MetS) individuals was previously described [24]. The most significant mRNA signature of the module organized around 18:1(cis-9) (degree: 38; betweenness centrality p-value = 0.85—not significant) was “Fatty Acid Biosynthesis”. Transcripts of this path included all desaturases (*SCD*, *FADS1* and *FADS2*), *ALDH6A1* and *ACSL1*. Like *AACS* and *LPIN1*, two other transcripts involved in lipid metabolism, all transcripts but *ALDH6A1* were positively and negatively connected to 14:1(cis-9) and 18:1(cis-9), respectively.

**Figure 4:**
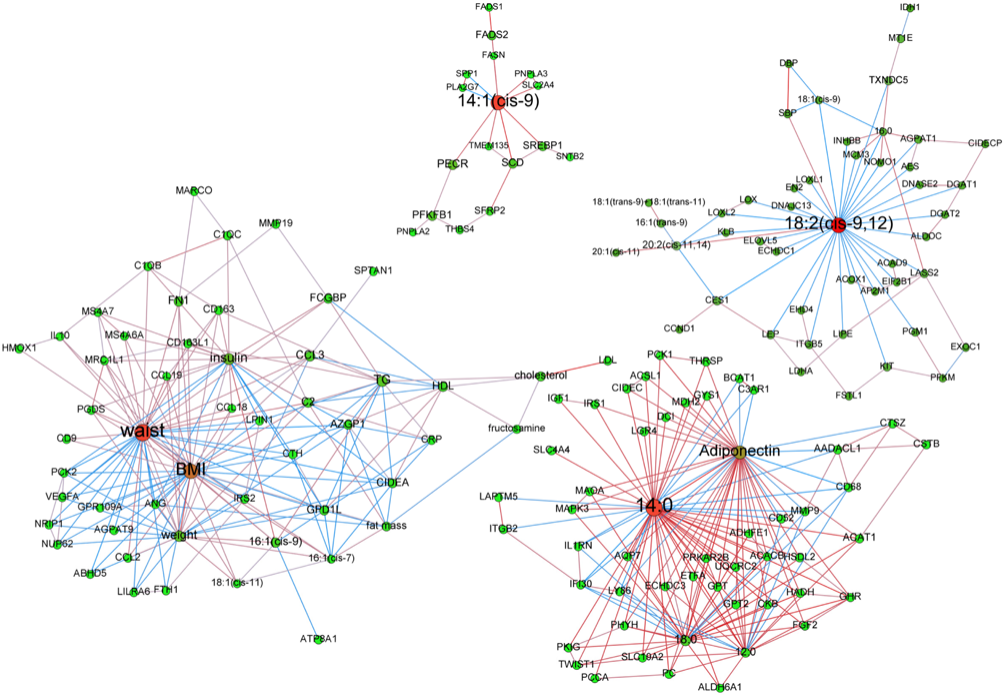
Adipose tissue networks of obese women after a 8-week low calorie diet. A sparse Graphical Gaussian Model (GGM) was used to estimate partial correlations in each set of variables and regularized canonical correlation analysis (CCA) was used to assess links between paired sets of variable. Clustering was performed using a spin glass model and simulated annealing. This analysis displays the variables that are connected independently from other variables. Graphs were laid out using force-based algorithms in Gephi 0.8.2 software. Nodes’ colors and font size indicate betweenness centrality. The red nodes have the highest betweenness and the green nodes the lowest one. Edge thickness is proportional to the strength of the correlation (CCA) or of the partial correlation (GGM). Edge color indicates the correlation sign: red for positive correlations and blue for negative ones. BMI, body mass index; CRP, C reactive protein; DBP, diastolic blood pressure; HDL, high density lipoprotein; LDL, low density lipoprotein; SBP, systolic blood pressure; TG, plasma triglycerides; waist, waist circumference.

**Effect of an 8-week active weight loss**. After LCD, vertex clustering detected 10 modules of which 7 had more than 6 nodes. Fig. 4 displays the 4 modules with at least 2 types of variables. Hubs were 14:0 (degree: 49; betweenness centrality p-value < 0.01), waist circumference (degree: 38; betweenness centrality p-value: 0.98—not significant), 14:1(cis-9)) (degree: 10; betweenness centrality p-value < 0.01), and 18:2(cis-9, 12) (degree: 34; betweenness centrality p-value: 0.38—not significant).

The 14:0 centered module also contained adiponectin as central node. The most significant mRNA signature was “Growth Hormone Signaling”. The transcripts from this signature (*GHR, IGF1, IRS1*, and *MAPK3)* were all positively connected to 3 saturated FAs, *i*.*e*. 12:0, 14:0 or 18:0 as well as to adiponectin. The module with waist circumference as hub also included BMI as high degree (35) and high centrality node. The most significant mRNA signature was “Adhesion and Diapedesis”. Transcripts from this signature (*CCL2, CCL3, CCL18, CCL19, FN1* and *MMP19*) were all positively connected to waist circumference, except *MMP19*. *CCL3* was positively connected to waist circumference whereas *GPD1L* and *AZGP1* were negatively connected to this abdominal adiposity marker. In this module, 16:1(cis-9) was negatively connected to *GPD1L* and positively to anthropometric parameters, plasma triglycerides and insulin. The module with highest degree node 14:1(cis-9) encompassed genes involved in “Fatty Acid Biosynthesis” (*SCD*, *FADS1* and *FADS2*) as well as *SLC2A4*, *FASN*, *SREBP1*, *PNPLA2* and *PNPLA3* in a positive manner. Of note, all of these genes were significantly down-regulated during LCD. The 18:2(cis-9, 12) with highest degree node mostly contained transcripts with negative relationship to this FA. These transcripts included those encoding proteins involved in triglyceride metabolism (*LIPE*, *DGAT1*, *DGAT2* and *AGPAT1*).

**Figure 5:**
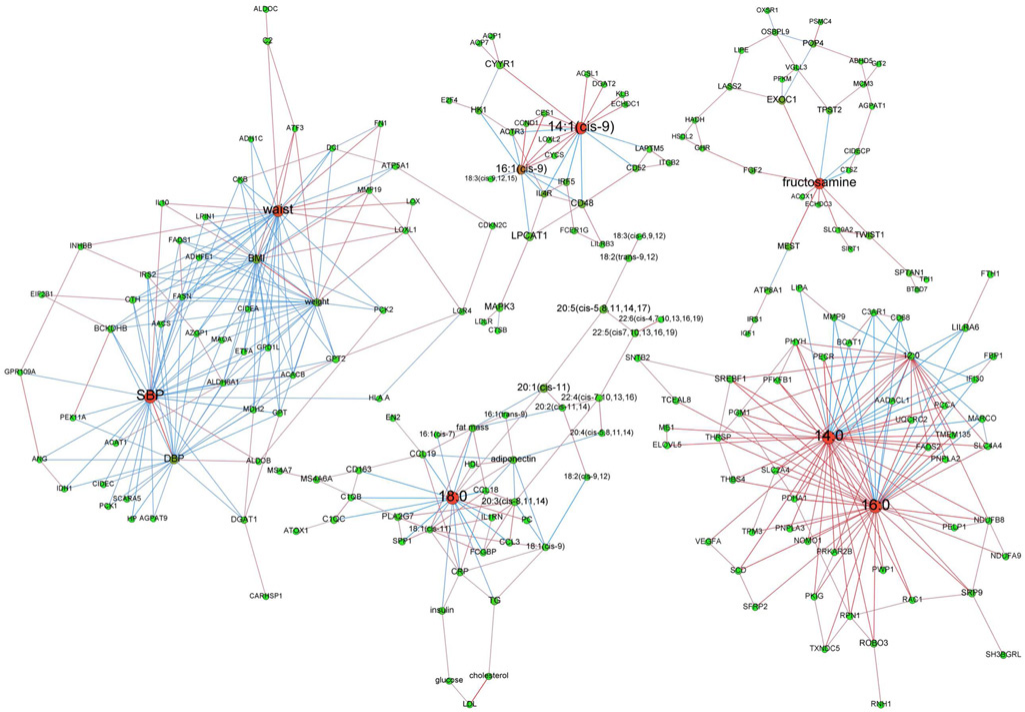
Adipose tissue networks during maintenance phase in women regaining weight. A sparse Graphical Gaussian Model (GGM) was used to estimate partial correlations in each set of variables and regularized canonical correlation analysis (CCA) was used to assess links between paired sets of variable. Clustering was performed using a spin glass model and simulated annealing. This analysis displays the variables that are connected independently from other variables. Graphs were laid out using force-based algorithms in Gephi 0.8.2 software. Nodes’ colors and font size indicate betweenness centrality. The red nodes have the highest betweenness and the green nodes the lowest one. Edge thickness is proportional to the strength of the correlation (CCA) or of the partial correlation (GGM). Edge color indicates the correlation sign: red for positive correlations and blue for negative ones. BMI, body mass index; CRP, C reactive protein; DBP, diastolic blood pressure; HDL, high density lipoprotein; LDL, low density lipoprotein; SBP, systolic blood pressure; TG, plasma triglycerides; waist, waist circumference.

**Figure 6:**
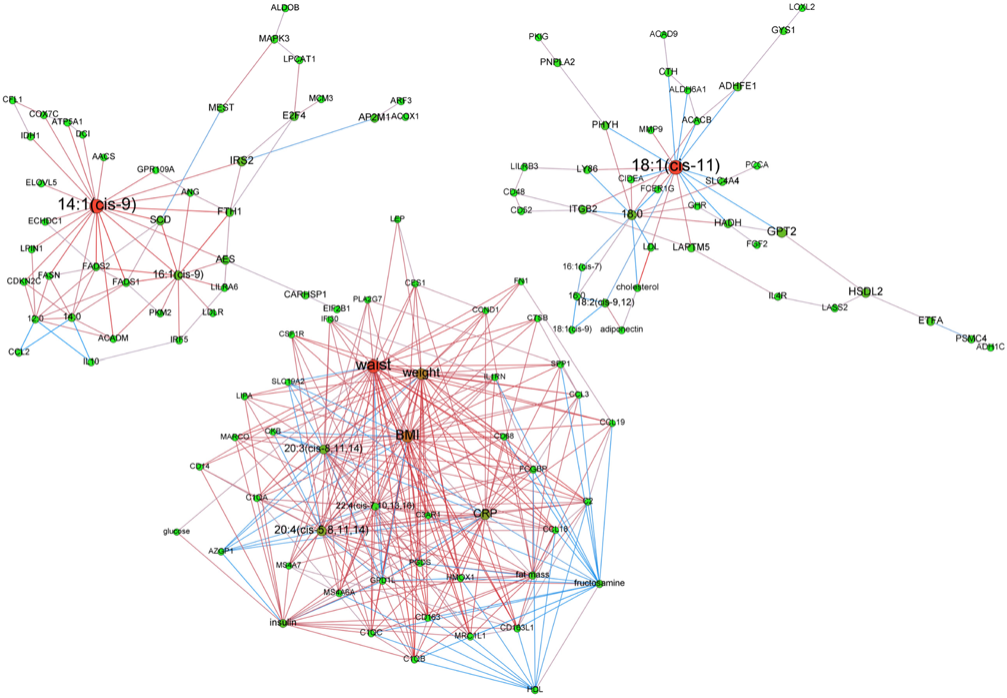
Adipose tissue networks during maintenance phase in women with continued weight loss. A sparse Graphical Gaussian Model (GGM) was used to estimate partial correlations in each set of variables and regularized canonical correlation analysis (CCA) was used to assess links between paired sets of variable. Clustering was performed using a spin glass model and simulated annealing. This analysis displays the variables that are connected independently from other variables. Graphs were laid out using force-based algorithms in Gephi 0.8.2 software. Nodes’ colors and font size indicate betweenness centrality. The red nodes have the highest betweenness and the green nodes the lowest one. Edge thickness is proportional to the strength of the correlation (CCA) or of the partial correlation (GGM). Edge color indicates the correlation sign: red for positive correlations and blue for negative ones. BMI, body mass index; CRP, C reactive protein; DBP, diastolic blood pressure; HDL, high density lipoprotein; LDL, low density lipoprotein; SBP, systolic blood pressure; TG, plasma triglycerides; waist, waist circumference.

**After 6 months weight maintenance diet**. Vertexes classification was performed and the most important heterogeneous clusters with more than 6 nodes are presented in Figs. 5 and 6. Since WS group showed intermediary phenotype, we focused on WR and WL groups.

**Individuals regaining weight**. Of 12 modules, classification detected 5 heterogeneous clusters of interest. As displayed in Fig. 4, the systolic blood pressure (degree: 34; betweenness centrality p-value = 0.05) and waist circumference (degree: 32; betweenness centrality p-value = 0.33—not significant) hubs showed negative relationship of these nodes with a “Sucrose, Serotonin and Adrenalin Degradation” IPA signature made of *ADHFE1*, *ALDOB, ALDOC, C2* and *MAOA*. These central nodes were also negatively connected to *AZGP1* and *GPD1L* and positively to *IL10*. The module converging on fructosamine (degree: 10; betweenness centrality p-value = 0.12—not significant) showed no FA but a “Growth Hormone Signaling” mRNA signature that included *IRS1*, *FGF2*, *IGF1* and *GHR*, the 2 former transcripts being significantly up-regulated during WMD in the WR group and the latter positively connected to fructosamine via *FGF2*. In the module organized around 14:1(cis-9) (degree: 16; betweenness centrality p-value = 0.62—not significant) the most significant mRNA signature was “Cancer Signal” mainly represented by transcripts up-regulated during WMD, *i*.*e*. *CCND1*, *CYCS*, *E2F4*, *ITGB2*, and *MAPK3*. 16:1(cis-9) was another hub (degree: 12) positively connected to 14:1(cis-9).

Two modules were with saturated FAs as hubs. The first one focused on 18:0 (degree: 20; betweenness centrality p-value = 0.09—not significant) and contained a large array of poly-unsaturated FAs plus 18:1(cis-9) which amount significantly decreased during WMD, exclusively in WR group. The most significant mRNA signature was “Adhesion and Diapedesis” (*CCL18, CCL19, CCL3* and *IL1RN*). The second cluster was organized around 14:0 (degree: 42; betweenness centrality p-value = 0.66—not significant) which was positively connected to 16:0 and 12:0. The most significant mRNA signature was “Angiogenesis Inhibition by TSP1”, especially *VEGFA*, an mRNA up-regulated during WMD and positively connected to 14:0, and *MMP9* with negative relationship to 14:0. The *SCD*, *FADS2*, *ELOVL5* and *SREBP1* transcripts involved in DNL were positively connected to 12:0, 14:0 or 16:0.

**Individuals with continued weight loss**. Of 11 modules, the 3 heterogeneous clusters are presented in Fig. 5. The 14:1(cis-9) centered module (degree: 21; betweenness centrality p-value = 0.01) encompassed genes involved in DNL, *i*.*e*. *AACS*, *FASN, SCD*, *FADS1, FADS2* and *ELOVL5*. All were positively correlated to 14:1(cis-9). The *AACS*, *SCD*, *FADS1* and *ELOVL5* mRNA levels increased during WMD. The most complex path was based on waist circumference (degree: 38; betweenness centrality p-value = 0.23—not significant) and incorporates BMI, weight, C reactive protein (CRP) and 20:4(cis-5, 8, 11, 14) as nodes with high centrality. *AZGP1* and *GPD1L* were negatively connected to waist circumference. The most significant mRNA signature was “Complement Adhesion and Diapedesis”. This included *CCL3, CCL18* and *CCL19*, *C1QA*, *C1QB* and *C1QC* that displayed significantly decreased mRNA levels during WMD and positive correlation with waist circumference. All FAs were n-6 with positive relationship to waist circumference, weight and BMI. Especially, the 20:4(cis-5, 8, 11, 14) had positive correlation to CRP. The module organized around 18:1(cis-11) (degree: 17; betweenness centrality p-value = 0.11—not significant) contained low density lipoproteins, cholesterol and adiponectin but showed no enriched mRNA signature.

## Discussion

Both lipids and transcripts (as frames for protein synthesis) are important components of AT biology. To identify interactions between these molecular species we investigated the networks of AT esterified FAs and mRNAs together with bio-clinical data in obese women according to weight changes along a longitudinal DI. The present study is the first to jointly investigate gene expression and lipidome from the same biopsy of AT in such a large number of obese individuals.

Networks are useful models to investigate a set of relations between variables. In particular, network clusters in gene networks are more robust, *i*.*e*., less influenced by measurement noise, than each individual relation [15, 40]. In the present case, the strength of the relations between the different sets of variables (*e*.*g*., the strength of the relation between two transcripts or the strength of the relation between a transcript and a FA level) have very different scales. This caveat is controlled using a non-global inference approach, in order to have a global model of the interactions between all sets of variables. The regularized CCA has previously been used in combination with sparse partial least squares regression to investigate AT transcriptionally coordinated paths correlated with PUFA intake during the LIPGENE study [17]. Here, we used a 3-step inference method to infer a global model using 3 datasets: first, inferring a network in each dataset using a sparse GGM; second, inferring a network between each pairs of two different sets of variables using regularized CCA; third, merging the 3 networks obtained during the first step with the 3 networks resulting from the second step. As the numbers of variables in the 3 datasets were very different (from 15 bio-clinical variables up to 221 mRNA levels), a simple strategy consisting in estimating the selected edges in each set of variables (or in each pair of two sets) in a same manner would have led to give too much importance on the largest set of variables, *i*.*e*., to the gene expression dataset. The number of selected edges was thus adjusted to be equal to the number of nodes in each set (or pair of sets) of variables, leading to smaller densities for the largest networks. To improve the significance of our findings, systematic statistical tests were performed to test the significance of the betweenness centrality of the nodes compared to their degrees. Significance of nodes indicates that, given their degrees, they have a betweenness larger than expected and are thus significantly central in their clusters.

Our study showed both constant and specific biological signatures in response to different weight control phases relevant to distinct metabolic features. We focused on body weight changes and especially according to weight control 6 months after calorie restriction. The present combination of network inference and node clustering enabled to draw a picture of transcript-FA-bioclinical variables interactions at each step of the longitudinal dietary intervention, leading to highlight the unexpected pivotal position of myristoleic acid (14:1(cis-9)). This FA was linked to DNL transcripts during active and continued weight loss. It is to be noted that, after WMD, the WR group merely displayed specific AT signatures never found at baseline or during weight loss.

The most striking invariable feature was the presence of waist circumference as central node along all steps of the DI. To check similarity between all clusters with waist circumference as hub, paired comparison of the number of common nodes between clusters was performed between baseline cluster and either LCD, or WL group, or WR group cluster. The p-values of these tests were all < 0.001, indicating a high similarity between nodes’ composition of the clusters having waist circumference for hub. Waist circumference is the most prominent clinical risk factor involved in MetS [41]. A persistent positive link with the macrophage inflammatory protein 1α (CCL3) and negative with the adipokine α2-glycoprotein 1 (AZGP1) and the enzyme glycerol-3-phosphate dehydrogenase 1-like (GPD1L) mRNA levels was found at baseline, after active weight loss and at 6 months of the weight control follow-up in WL and WR groups. Variants in *GPD1L* are associated with risk of sudden death in patients with coronary artery disease [42]. AZGP1 is a lipid mobilizing factor with putative role in insulin resistance as mRNA and protein were low in AT of type 2 diabetes patients and circulating AZGP1 protein inversely correlated with BMI and waist-to-hip ratio [43]. The chemokine CCL3 is up-regulated with insulin resistance in AT [44]. These genes are at top rank of the MetS signature described in AT from obese individuals [24]. The relationship between these transcripts and the major component of MetS suggests that they could be used as biomarkers for risk stratification of type 2 diabetes or cardiovascular disease in obese individuals, alone or combined to bio-clinical related factors.

At baseline, fasting plasma insulin was the most significant central vertex among all modules. This cluster exhibited an immune signature, all transcripts of the Adhesion and Diapedesis pathway being positively connected to insulin. On the other hand, insulin was negatively connected to stearic acid and transcripts encoding factors involved in lipid metabolism (*CIDEA*) [45], especially lipolysis (*GPR109A* and *ABDH5*) [46], and *SIRT1*. The *SIRT1* gene encodes a histone deacetylase that regulates various metabolic pathways and regulate lipids and glucose metabolism [47]. Besides the positive relationship between immune cells content in AT in the etiology of insulin resistance [48], this cluster indicates that, in obese women, the higher is the insulin level at fasting, the lower is the lipid metabolism signaling in AT.

After LCD induced weight loss, 3 modules focused on FAs. One was organized around linoleic acid, an essential FA that is highly represented (>30%) in the commercial hypocaloric meals provided during LCD (data not shown). However, linoleic acid (18:2(cis-9, 12)) content of fat pads was unchanged compared to baseline. Indeed, there is minimal deposition of dietary fat into AT during periods of negative energy balance [9]. Myristic acid (14:0) was the most central vertex of a module along with lauric (12:0) and stearic acids (18:0). Adiponectin, which is an adipocytokine with anti-inflammatory and insulin sensitive properties [49] was another central vertex. Myristic acid and adiponectin were both positively connected between each other and to insulin signaling or insulin-like transcripts (*IRS1* and *IGF1*). The biological role of myristic acid remains poorly explored. Fatty acylation of signaling proteins play key roles in regulating cellular structure and function. Among the various myristoylated proteins are numerous signal transducing proteins [50]. In the present study, there was a statistically significant decrease in myristic acid triglycerides AT content during LCD, indicating a mobilization from lipid droplet that might provide non esterified myristic acid within the adipose cell. Whether such available myristic acid indeed does acylate signal transduction proteins is a question of particular interest.

Six months after the end of LCD, the AT from women that continued to lose weight (WL group) also displayed two modules organized around FAs, myristoleic acid and vaccenic acid. Vaccenic acid amount is low in AT (<2%). It comes from palmitoleic acid elongation. There was no significant change in AT vaccenic acid content during the dietary intervention. Its steadiness in AT from individuals continuing to lose weight indicates that this FA was poorly mobilized during weight loss. A positive correlation between vaccenic plasma TG content and insulin resistance has been shown in men [51]. Whether there is a similar link with AT triglycerides deserves attention even though no direct relationship with glucose homeostasis parameters appears in the present module.

When considering active weight loss and continued weight loss after calorie restriction, a remarkable feature was the presence of myristoleic acid connected to an array of genes involved in FA synthesis, especially DNL enzymes and desaturases (*FASN*, *SCD*, *FADS1* and *FADS2*). Like palmitoleic (16:1(cis-9)) and oleic acids (18:1(cis-9)), myristoleic acid is a product of desaturation by SCD (from myristic acid). It is a minor AT FA (<0.5% of total FA content) that is not provided by food. Surprisingly, in the present study it is an important focal node, which AT content decreased during LCD and remained stable at the end of WMD, except in WR group. Moreover, at the end of WMD, in WR group and in relation to *SCD* gene expression in AT, an increased SCD activity (assessed by 14:1(cis-9)/14:0 ratio) was observed that could be due to a positive regulation of SCD transcription by saturated FAs [52]. In this group, 14:0 and 16:0 were focal nodes and positively connected to *SCD*. The SCD activity is known to be associated with triglyceride accumulation [53] but its beneficial effect on insulin sensitivity remains controversial [52]. Control of *SCD* expression and DNL are coordinated. SCD is tightly regulated by saturated FAs and poly-unsaturated FAs as linoleic acid, SREBP-1c and carbohydrate response element binding protein (ChREBP) α and β [52]. *ChREBP* isoforms were not included in the series of mRNA quantified here but *SREBP1* was positively connected to myristoleic acid after LCD. This is in agreement with the transcriptional activation of SCD by SREBP1c [52]. In contrast to liver where DNL is considered deleterious, DNL occurring in fat depots may provide beneficial health effects since it produces lipid species with bioactivities distinct from those of lipids predominantly derived from diet [2, 54]. Strategies to enhance DNL specifically in AT may provide new therapies for metabolic and cardiovascular diseases [55–57]. The presence of a DNL signature with acute (LCD) and continuing (WMD) weight loss is in line with the enhanced differentiation potential of preadipocytes observed after calorie restriction [58]. In the present study, myristoleic acid might be an interesting marker of DNL and SCD activity in AT. Its persistence in AT triglycerides despite fat mass loss may constitute a hallmark of beneficial adipogenesis after weight loss.

Last, AT from WR group showed a salient hyperplastic attribute with 3 modules encompassing genes involved in cell proliferation, angiogenesis, or growth factor signal transduction. Of note, the former cluster exhibited two mono-unsaturated FAs as central nodes with no link to genes involved in FA metabolism. The angiogenic signature was mainly due to *VEGFA* (mean fold change during WMD = 1.9±0.4) that encodes an essential proangiogenic factor in AT [59]. The latter was organized around fructosamine, which is a serum marker of poor long-term glycemic control, as a hallmark of the deleterious effect of energy store repletion. The positive link of fructosamine to a series of transcripts- *TWIST1* that encodes a transcription factor abundantly expressed in adipocytes [60], which is positively correlated to insulin sensitivity [61] and *SPTAN1*, a transcript encoding an insulin responsive α-fodrin involved in the glucose transporter GLUT4 translocation in adipocytes [62]-related to glucose homeostasis and beyond insulin signaling (*IRS1*, *IGF1, FGF2* and *GHR*) may seem counterintuitive. Growth hormone shares protein anabolic properties with insulin. On the other hand, fasting insulin and glucose are part of another module which displays an immune signature (Adhesion and Diapedesis), emphasizing the link between adipose tissue inflammatory status and insulin resistance [48]. The link between weight regain and proliferative patterns was previously shown using transcriptomic in a small subset of individuals from the same trial [28]. The present study indicates AT hyperplasia in individuals failing weight maintenance despite continued energy restriction. Altogether, no cluster showed a lipid metabolism signature in this group. Stearic acid was the hub of a module with the immune response signature. This FA was negatively connected to unfavorable bio-clinical parameters (body fat mass, fasting plasma insulin, triglycerides and CRP) and positively to beneficial ones (adiponectin and HDL). This suggests that the highest is lipid droplet stearic acid content, the better is metabolic status. The cluster with systolic blood pressure and waist circumference as hubs displayed an amine degradation signature. Levels of noradrenaline associate with obesity and cardiovascular risk [63]. Systolic blood pressure was negatively correlated to most variables, including *AZGP1* and *GPD1L* described above except diastolic blood pressure. This parameter was negatively correlated to waist circumference as well as BMI. This feature was different from the one observed in the weight loss group where waist circumference was positively correlated to weight and BMI. This emphasizes the predominant role of waist circumference, compared to blood pressure, in metabolic syndrome compared to blood pressure.

The present investigation shows several limitations. Only women were investigated; as a preeminent effect of sex on AT gene expression was previously shown [24, 64]. Also, we studied fat from the subcutaneous abdominal region and we cannot extrapolate our findings to other subcutaneous, gluteo-femoral or visceral fat depots. Last, we performed unsupervised learning using GGM. This approach uses partial correlations and differs from relevance networks that use direct correlations and thus provide a strong but sometimes biased measure of the dependence between variables. Bayesian networks that lead to directed acyclic graphs (DAG) could provide a clue on causal relationships but some knowledge information has to be provided *a priori*. In the present networks, edges do not represent simple correlations but between variables dependencies. Using GGM, interpretation is not causality but only a matter of strong and direct statistical association. Nodes with highest betweenness centrality represent variables whose fine tuning might greatly impact the level of the other connected variables.

To conclude, this approach has linked a characteristic structure of AT network to a slimmed phenotype thereby suggesting myristoleic acid as main lipidic biomarker for DNL and SCD activity. The anabolic signature unique to individuals with unsuccessful weight control suggests detrimental tissue hyperplasia. This initial analysis provides a valuable starting point for more in-depth investigation of the implication of myristoleic acid in weight loss.

## Supporting Information

S1 Protocol Detailed study protocol of the DiOGenes trial. (PDF)

S1 Checklist CONSORT checklist of the DiOGenes trial. (DOC)

S1 Table Fatty acids extracted from the human adipose tissue biopsies lipid fraction. (DOCX)

S2 Table Description of the target genes investigated. (DOCX)

S3 Table Anthropometric and clinical characteristics at the end of the weight maintenance phase according to weight control group and by randomization arm. (DOCX)

S4 Table Adipose tissue fatty acid content according to weight control groups during dietary intervention. (DOCX)

S1 Fig Energy and fat dietary intake changes according to weight control groups during dietary intervention. Energy intake (A), fat (B), protein (C), and carbohydrate intake (D) were investigated by a nutritionist using a weighed food record at screening, four weeks (week 4) after the end of low calorie diet (LCD) and at the end of weight maintenance diet (WMD). At LCD, energy intake, fat, protein and carbohydrate intake data issued from commercial meal replacement information. Triangles/black lines represent weight loss group; squares/grey lines represent weight stable group and circles/dotted lines represent weight regain group. Variables are shown as means ± SEM. Δ: p < 0.05, data different in weight loss group, #: p < 0.05, data different in weight stable group, ●: p < 0.05, data different in weight regain group.
(TIF)

S2 Fig Profiles of the most varying adipose tissue mRNAs according to weight control groups during dietary intervention. Adipose tissue mRNA levels of SCD (A) and FASN (B) were determined according to groups at baseline (BAS), after 8 weeks low calorie diet (LCD), and after 6 months of weight maintenance diet (WMD). Triangles/black lines represent weight loss group; squares/grey lines represent weight stable group and circles/dotted lines represent weight regain group. Variables are shown as means ± SEM. Δ: p < 0.05, data different in weight loss group.; #: p < 0.05, data different in weight stable group.; ●: p < 0.05, data different in weight regain group.; §: p < 0.05, data difference between groups.
(TIF)

S3 Fig Stearoyl coA desaturase activities according to weight control groups during dietary intervention. Stearoyl coA desaturase (SCD) activities were assessed using C14:1 n-5/C14:0, C16:1 n-7/C16:0 and C18:1 n-9/C18:0 ratios and plotted according to groups at baseline (BAS), after 8 weeks low calorie diet (LCD), and after 6 months of weight maintenance diet (WMD). Triangles/black lines represent weight loss group; squares/grey lines represent weight stable group and circles/dotted lines represent weight regain group. Variables are shown as means ± SEM. Δ: p < 0.05, data different in weight loss group; #: p < 0.05, data different in weight stable group; •: p < 0.05, data different in weight regain group; §: p < 0.05, data difference between groups.
(TIF)
