# Supplementary material for: System Model Network for Adipose Tissue Signatures Related to Weight Changes in Response to Calorie Restriction and Subsequent Weight Maintenance
Source: PLoS Comput Biol. 2015 Jan 15;11(1):e1004047. doi: 10.1371/journal.pcbi.1004047 (PMC4295881; doi:10.1371/journal.pcbi.1004047)
Supplement: S1 Table — (DOCX) [file pcbi.1004047.s003.docx]

**Table S1**. Fatty acids from the human adipose tissue lipid fraction.

|  | |  |  | |  |  |
| --- | --- | --- | --- | --- | --- | --- |
|  | | Numerical symbol | | Systematic name | Trivial name | |
| **SFAs** | |  | |  |  | |
|  | | 12:0 | | dodecanoic acid | lauric acid | |
|  | | 14:0 | | tetradecanoic acid | myristic acid | |
|  | | 16:0 | | hexadecanoic acid | palmitic acid | |
|  | | 18:0 | | octadecanoic acid | stearic acid | |
|  | | 20:0 | | eicosanoic acid | arachidic acid | |
| **MUFAs** | |  | |  |  | |
|  | | 14:1(cis-9) | | cis-9-tetradecenoic acid | myristoleic acid | |
|  | | 16:1(cis-9) | | cis-9-hexadecenoic acid | palmitoleic acid | |
|  | | 16:1(trans-9) | | trans-9-hexadecenoic acid | palmitelaidic acid | |
|  | | 16:1(cis-7) | | cis-7-hexadecenoic acid |  | |
|  | | 18:1(cis-11) | | cis-11-octadecenoic acid | vaccenic acid | |
|  | | 18:1(cis-9) | | cis-9-octadecenoic acid | oleic acid | |
|  | | 18:1(trans-9) + 18:1(trans-11) | | trans-9-octadecenoic acid + trans-11-octadecenoic acid |  | |
|  | | 20:1(cis-11) | | cis-9-eicosenoic acid | gondoic acid | |
| **PUFAs** | |  | |  |  | |
|  | | 18:2(cis-9,12) | | cis-9, 12-octadecadienoic acid | linoleic acid | |
|  | | 18:2(trans-9,12) | | trans-9, 12-octadecadienoic acid | linolenelaidic acid | |
|  | | 18:2(trans-9,cis-12) | | trans-9, cis-12-octadecadienoic acid |  | |
|  | | 18:2(cis-9,trans-12) | | cis-9, trans-12-octadecadienoic acid |  | |
|  | | 18:3(cis-6,9,12) | | cis-6, 9, 12-octadecatrienoic acid | γ-linolenic acid | |
|  | | 18:3(cis-9,12,15) | | cis-9, 12, 15-octadecatrienoic acid | linolenic acid | |
|  | | 20:2(cis-11,14) | | cis-11, 14-eicosadienoic acid |  | |
|  | | 20:3(cis-5,8,11) | | cis-5, 8, 11-eicosatrienoic acid |  | |
|  | | 20:3(cis-8,11,14) | | cis-8, 11, 14-eicosatrienoic acid |  | |
|  | | 20:4(cis-5,8,11,14) | | cis-5, 8, 11, 14-eicosatetraenoic acid | arachidonic acid | |
|  | | 20:5(cis-5,8,11,14,17) | | cis-5,8,11,14,17-eicosapentaenoic acid |  | |
|  | | 22:4(cis-7,10,13,16) | | cis-7, 10, 13, 16-docosatetraenoic acid |  | |
|  | | 22:5(cis-4,7,10,13,16) | | cis-4,7, 10, 13, 16-docosapentaenoic acid |  | |
|  | | 22:5(cis-7,10,13,16,19) | | cis-7, 10, 13, 16, 19-docosapentaenoic acid |  | |
|  | | 22:6(cis-4,7,10,13,16,19) | | cis-4, 7, 10, 13, 16, 19-docosahexaenoic acid |  | |

The numerical symbol is presented according to the International Union of Pure and Applied Chemistry (IUPAC) nomenclature.

SFAs, saturated fatty acids; MUFAs, mono unsaturated fatty acids; PUFAs, poly unsaturated fatty acids.
