# Supplementary material for: System Model Network for Adipose Tissue Signatures Related to Weight Changes in Response to Calorie Restriction and Subsequent Weight Maintenance
Source: PLoS Comput Biol. 2015 Jan 15;11(1):e1004047. doi: 10.1371/journal.pcbi.1004047 (PMC4295881; doi:10.1371/journal.pcbi.1004047)
Supplement: S2 Table — (DOCX) [file pcbi.1004047.s004.docx]

**TABLE S2.** Description of the target genes investigated.

| Gene Symbol | Gene Name | Biological Function |
| --- | --- | --- |
| **Amino Acid Metabolism** | | |
| *ALDH6A1* | aldehyde dehydrogenase 6 family, member A1 | amino acid catabolism |
| *BCAT1* | branched chain aminotransferase 1, cytosolic | amino acid catabolism |
| *BCKDHB* | branched chain keto acid dehydrogenase E1, beta polypeptide | amino acid catabolism |
| *CTH* | cystathionase (cystathionine gamma-lyase) | amino acid synthesis |
| *GPT* | glutamic-pyruvate transaminase (alanine aminotransferase) | amino acid synthesis |
| *GPT2* | glutamic pyruvate transaminase (alanine aminotransferase) 2 | amino acid synthesis |
| **Carbohydrate Metabolism** | | |
| *ADH1C* | alcohol dehydrogenase 1C (class I), gamma polypeptide | aerobic glycolysis |
| *ALDOB* | aldolase B, fructose-bisphosphate | aerobic glycolysis |
| *ALDOC* | aldolase C, fructose-bisphosphate | aerobic glycolysis |
| *ENO1* | enolase 1, (alpha) | aerobic glycolysis |
| *ENO3* | enolase 3 (beta, muscle) | aerobic glycolysis |
| *FBP1* | fructose-1,6-bisphosphatase 1 | aerobic glycolysis |
| *GAPDH* | glyceraldehyde-3-phosphate dehydrogenase | aerobic glycolysis |
| *GYS1* | glycogen synthase 1 (muscle) | glycogenesis |
| *HK1* | hexokinase 1 | aerobic glycolysis |
| *LDHA* | lactate dehydrogenase A | anaerobic glycolysis |
| *PC* | pyruvate carboxylase | gluconeogenesis |
| *PCK1* | phosphoenolpyruvate carboxykinase 1 (soluble) | aerobic glycolysis |
| *PCK2* | phosphoenolpyruvate carboxykinase 2 (mitochondrial) | aerobic glycolysis |
| *PDHA1* | pyruvate dehydrogenase (lipoamide) alpha 1 | aerobic glycolysis |
| *PFKFB1* | 6-phosphofructo-2-kinase/fructose-2,6-biphosphatase 1 | aerobic glycolysis |
| *PFKM* | phosphofructokinase, muscle | aerobic glycolysis |
| *PGAM1* | phosphoglycerate mutase 1 (brain) | aerobic glycolysis |
| *PGK1* | phosphoglycerate kinase 1 | aerobic glycolysis |
| *PGM1* | phosphoglucomutase 1 | aerobic glycolysis |
| *PKM2* | pyruvate kinase, muscle | aerobic glycolysis |
| *SLC2A4* | solute carrier family 2 (facilitated glucose transporter), member 4 | glucose transport |
| *TPI1* | triosephosphate isomerase 1 | aerobic glycolysis |
| **Cell Differentiation** | | |
| *CES1* | carboxylesterase 1 (monocyte/macrophage serine esterase 1) | adipogenesis |
| *FSTL1* | follistatin-like 1 | cell differenciation |
| *INHBB* | inhibin, beta B | energy balance |
| *MEST* | mesoderm specific transcript homolog (mouse) | adipogenesis |
| *PEX11A* | peroxisomal biogenesis factor 11 alpha | peroxysome organization |
| *ROBO3* | roundabout, axon guidance receptor, homolog 3 (Drosophila) | cell differenciation |
| *SFRP2* | secreted frizzled-related protein 2 | adipogenesis |
| **Cell Proliferation** | | |
| *ANG* | angiogenin, ribonuclease, RNase A family, 5 | angiogenesis |
| *CCND1* | cyclin D1 | cell cycle |
| *CDK2AP1* | cyclin-dependent kinase 2 associated protein 1 | cell cycle |
| *CDKN2C* | cyclin-dependent kinase inhibitor 2C (p18, inhibits CDK4) | cell cycle |
| *DNASE2* | deoxyribonuclease II, lysosomal | apoptosis |
| *FGF2* | fibroblast growth factor 2 (basic) | cell cycle |
| *GHR* | growth hormone receptor | lipolysis |
| *IGF1* | insulin-like growth factor 1 (somatomedin C) | glycogen synthesis |
| *IRS1* | insulin receptor substrate 1 | insulin signaling |
| *IRS2* | insulin receptor substrate 2 | insulin signaling |
| *MAPK3* | mitogen-activated protein kinase 3 | cell cycle |
| *PRKAR2B* | protein kinase, cAMP-dependent, regulatory, type II, beta | signal transduction |
| *VEGFA* | vascular endothelial growth factor A | angiogenesis |
| **Cell Structure** | | |
| *ARPC1A* | actin related protein 2/3 complex, subunit 1A, 41kDa | cytoskeleton organization |
| *CFL1* | cofilin 1 (non-muscle) | cytoskeleton organization |
| *GIT2* | G protein-coupled receptor kinase interacting ArfGAP 2 | cytoskeleton organization |
| *SNTB2* | syntrophin, beta 2 (dystrophin-associated protein A1, 59kDa, basic component 2) | cytoskeleton organization |
| *SPTAN1* | spectrin, alpha, non-erythrocytic 1 (alpha-fodrin) | cytoskeleton organization |
| *TUBA1A* | tubulin, alpha 1a | cytoskeleton organization |
| *TPM3* | tropomyosin 3 | cytoskeleton organization |
| *WDR1* | WD repeat domain 1 | cytoskeleton organization |
| **Energy Metabolism** | | |
| *ADHFE1* | alcohol dehydrogenase, iron containing, 1 | ketone metabolism |
| *ATP5A1* | ATP synthase, H+ transporting, mitochondrial F1 complex, alpha subunit 1, cardiac muscle | oxydative phosphorylation |
| *ATP8A1* | ATPase, aminophospholipid transporter, class I, type 8A, member 1 | oxydative phosphorylation |
| *CKB* | creatine kinase, brain | creatin phosphate shuttle |
| *COX7C* | cytochrome c oxidase subunit VIIc | oxydative phosphorylation |
| *CYCS* | cytochrome c, somatic | oxydative phosphorylation |
| *ETFA* | electron-transfer-flavoprotein, alpha polypeptide | fatty acid oxidation |
| *ETFDH* | electron-transferring-flavoprotein dehydrogenase | fatty acid oxidation |
| *GATM* | glycine amidinotransferase (L-arginine:glycine amidinotransferase) | creatin biosynthesis |
| *IDH1* | isocitrate dehydrogenase 1 (NADP+), soluble | tricarboxylic acid cycle |
| *LEP* | leptin | food intake control |
| *MDH2* | malate dehydrogenase 2, NAD (mitochondrial) | tricarboxylic acid cycle |
| *ME1* | malic enzyme 1, NADP(+)-dependent, cytosolic | tricarboxylic acid cycle |
| *NDUFA9* | NADH dehydrogenase (ubiquinone) 1 alpha subcomplex, 9, 39kDa | oxydative phosphorylation |
| *NDUFB8* | NADH dehydrogenase (ubiquinone) 1 beta subcomplex, 8, 19kDa | oxydative phosphorylation |
| *UQCRC2* | ubiquinol-cytochrome c reductase core protein II | oxydative phosphorylation |
| **Immune Response** | | |
| *C1QA* | complement component 1, q subcomponent, A chain | complement system |
| *C1QB* | complement component 1, q subcomponent, B chain | complement system |
| *C1QC* | complement component 1, q subcomponent, C chain | complement system |
| *C2* | complement component 2 | complement system |
| *C3AR1* | complement component 3a receptor 1 | complement system |
| *CCL18* | chemokine (C-C motif) ligand 18 (pulmonary and activation-regulated) | chemotaxis |
| *CCL19* | chemokine (C-C motif) ligand 19 | immune response |
| *CCL2* | chemokine (C-C motif) ligand 2 | chemotaxis |
| *CCL3* | chemokine (C-C motif) ligand 3 | chemotaxis |
| *CD14* | CD14 molecule | response to LPS |
| *CD163* | CD163 molecule | acute phase response |
| *CD163L1* | CD163 molecule-like 1 | scavenger receptor activity |
| *CD48* | CD48 molecule | Immunoglobulin mediated immune response |
| *CD68* | CD68 molecule | scavenger receptor activity |
| *CD9* | CD9 molecule | immune response |
| *CSF1R* | colony stimulating factor 1 receptor | cell differentiation |
| *FCER1G* | Fc fragment of IgE, high affinity I, receptor for; gamma polypeptide | Immunoglobulin mediated immune response |
| *FCGBP* | Fc fragment of IgG binding protein | Immunoglobulin mediated immune response |
| *FN1* | fibronectin 1 | cell adhesion |
| *FTH1* | ferritin, heavy polypeptide 1 | iron homeostasis |
| *HLA-A* | major histocompatibility complex, class I, A | antigen presentation |
| *HMOX1* | heme oxygenase (decycling) 1 | hemoglobin degradation |
| *HP* | haptoglobin | hemoglobin degradation |
| *IFI30* | interferon, gamma-inducible protein 30 | antigen presentation |
| *IL10* | interleukin 10 | inflammatory cytokine |
| *IL1RN* | interleukin 1 receptor antagonist | inflammatory response |
| *IL4R* | interleukin 4 receptor | inflammatory response |
| *ITGB2* | integrin, beta 2 (complement component 3 receptor 3 and 4 subunit) | cell adhesion |
| *ITGB5* | integrin, beta 5 | cell adhesion |
| *KIT* | v-kit Hardy-Zuckerman 4 feline sarcoma viral oncogene homolog | immune response |
| *LILRA6* | leukocyte immunoglobulin-like receptor, subfamily A (with TM domain), member 6 | antigen presentation |
| *LILRB3* | leukocyte immunoglobulin-like receptor, subfamily B (with TM and ITIM domains), member 3 | inflammatory response |
| *LY86* | lymphocyte antigen 86 | inflammatory response |
| *MARCO* | macrophage receptor with collagenous structure | innate immune response |
| *MRC1L1* | mannose receptor, C type 1-like 1 | receptor-mediated endocytosis |
| *MS4A6A* | membrane-spanning 4-domains, subfamily A, member 6A | macrophage marker |
| *MS4A7* | membrane-spanning 4-domains, subfamily A, member 7 | macrophage marker |
| *NFKB2* | nuclear factor of kappa light polypeptide gene enhancer in B-cells 2 (p49/p100) | transcription |
| *PLA2G7* | phospholipase A2, group VII (platelet-activating factor acetylhydrolase, plasma) | inflammatory response |
| *RAC1* | ras-related C3 botulinum toxin substrate 1 (rho family, small GTP binding protein Rac1) | immune response |
| *SCARA5* | scavenger receptor class A, member 5 (putative) | iron transport |
| *SPP1* | secreted phosphoprotein 1 | chemotaxis |
| *THBS4* | thrombospondin 4 | cell adhesion |
| **Lipid Metabolism** | | |
| *AACS* | acetoacetyl-CoA synthetase | lipogenesis |
| *AADACL1* | neutral cholesterol ester hydrolase 1 | cholesterol metabolism |
| *ABHD5* | abhydrolase domain containing 5 | lipolysis |
| *ACACB* | acetyl-Coenzyme A carboxylase beta | lipogenesis |
| *ACAD9* | acyl-Coenzyme A dehydrogenase family, member 9 | lipogenesis |
| *ACADM* | acyl-Coenzyme A dehydrogenase, C-4 to C-12 straight chain | fatty acid oxydation |
| *ACAT1* | acetyl-Coenzyme A acetyltransferase 1 | lipogenesis |
| *ACOX1* | acyl-Coenzyme A oxidase 1, palmitoyl | fatty acid oxydation |
| *ACSL1* | acyl-CoA synthetase long-chain family member 1 | lipogenesis |
| *ACSS2* | acyl-CoA synthetase short-chain family member 2 | lipogenesis |
| *AGPAT1* | 1-acylglycerol-3-phosphate O-acyltransferase 1 (lysophosphatidic acid acyltransferase, alpha) | lipogenesis |
| *AGPAT9* | 1-acylglycerol-3-phosphate O-acyltransferase 9 | lipogenesis |
| *AQP1* | aquaporin 1 (Colton blood group) | glycerol transporter |
| *AQP7* | aquaporin 7 | glycerol transporter |
| *AZGP1* | alpha-2-glycoprotein 1, zinc-binding | lipolysis |
| *CIDEA* | cell death-inducing DFFA-like effector a | fatty acid oxydation |
| *CIDEC* | cell death-inducing DFFA-like effector c | lipogenesis |
| *DCI* | dodecenoyl-Coenzyme A delta isomerase (3,2 trans-enoyl-Coenzyme A isomerase) | fatty acid oxydation |
| *DGAT1* | diacylglycerol O-acyltransferase homolog 1 (mouse) | lipogenesis |
| *DGAT2* | diacylglycerol O-acyltransferase homolog 2 (mouse) | lipogenesis |
| *ECHDC1* | enoyl Coenzyme A hydratase domain containing 1 | fatty acid oxydation |
| *ECHDC3* | enoyl Coenzyme A hydratase domain containing 3 | fatty acid oxydation |
| *ELOVL5* | ELOVL family member 5, elongation of long chain fatty acids (FEN1/Elo2, SUR4/Elo3-like, yeast) | lipogenesis |
| *FADS1* | fatty acid desaturase 1 | lipogenesis |
| *FADS2* | fatty acid desaturase 2 | lipogenesis |
| *FASN* | fatty acid synthase | lipogenesis |
| *GPD1L* | glycerol-3-phosphate dehydrogenase 1-like | phospholipid metabolism |
| *GPR109A* | G protein-coupled receptor 109A | lipolysis |
| *HADH* | Hydroxyacyl-Coenzyme A dehydrogenase | fatty acid oxidation |
| *HSDL2* | hydroxysteroid dehydrogenase like 2 | lipolysis |
| *KLB* | klotho beta | cholesterol metabolism |
| *LASS2* | LAG1 homolog, ceramide synthase 2 | sphingolipid metabolism |
| *LDLR* | low density lipoprotein receptor | cholesterol metabolism |
| *LIPA* | lipase A, lysosomal acid, cholesterol esterase | cholesterol metabolism |
| *LIPE* | lipase, hormone-sensitive | lipolysis |
| *LPCAT1* | lysophosphatidylcholine acyltransferase 1 | phospholipid synthesis |
| *LPIN1* | lipin 1 | lipogenesis |
| *MECR* | mitochondrial trans-2-enoyl-CoA reductase | fatty acid oxydation |
| *OSBPL9* | oxysterol binding protein-like 9 | cholesterol transport |
| *PCCA* | propionyl Coenzyme A carboxylase, alpha polypeptide | fatty acid oxidation |
| *PECI* | peroxisomal D3,D2-enoyl-CoA isomerase | fatty acid oxidation |
| *PECR* | peroxisomal trans-2-enoyl-CoA reductase | fatty acid biosynthesis |
| *PGDS* | prostaglandin-H2 D-isomerase | prostaglandin metabolism |
| *PHYH* | phytanoyl-CoA 2-hydroxylase | fatty acid oxidation |
| *PKIG* | protein kinase (cAMP-dependent, catalytic) inhibitor gamma | lipolysis |
| *PNPLA2* | patatin-like phospholipase domain containing 2 (ATGL) | lipolysis |
| *PNPLA3* | patatin-like phospholipase domain containing 3 (adiponutrin) | lipolysis |
| *SCD* | stearoyl-CoA desaturase (delta-9-desaturase) | lipogenesis |
| *THRSP* | thyroid hormone responsive (SPOT14 homolog, rat) | lipogenesis |
| **Protein Metabolism** | | |
| *CST3* | cystatin C | tissue remodelling |
| *CSTB* | cystatin B (stefin B) | tissue remodelling |
| *CTSB* | cathepsin B | tissue remodelling |
| *CTSZ* | cathepsin Z | tissue remodelling |
| *LOX* | lysyl oxidase | tissue remodelling |
| *LOXL1* | lysyl oxidase-like 1 | tissue remodelling |
| *LOXL2* | lysyl oxidase-like 2 | tissue remodelling |
| *MMP19* | matrix metallopeptidase 19 | tissue remodelling |
| *MMP9* | matrix metallopeptidase 9 (gelatinase B, 92kDa gelatinase, 92kDa type IV collagenase) | tissue remodelling |
| *PSMC4* | proteasome (prosome, macropain) 26S subunit, ATPase, 4 | proteolysis |
| *RPN1* | ribophorin I | protein glycosylation |
| *TPST2* | tyrosylprotein sulfotransferase 2 | peptidyl-tyrosine sulfation |
| **Response to Stress** | | |
| *ATOX1* | ATX1 antioxidant protein 1 homolog (yeast) | copper ion transport |
| *MT1E* | metallothionein 1E | response to oxidative stress |
| *OXSR1* | oxidative-stress responsive 1 | response to oxidative stress |
| **Signal Transduction** | | |
| *ACTR3* | ARP3 actin-related protein 3 homolog (yeast) | endocytosis signaling |
| *AP2M1* | adaptor-related protein complex 2, mu 1 subunit | intracellular trafficking |
| *EHD4* | EH-domain containing 4 | endocytosis signaling |
| *LGR4* | leucine-rich repeat-containing G protein-coupled receptor 4 | membrane receptor |
| *MAOA* | monoamine oxidase A | dopamin catabolism |
| **Gene Expression** | | |
| *AES* | amino-terminal enhancer of split | transcription |
| *ATF3* | activating transcription factor 3 | transcription |
| *E2F4* | E2F transcription factor 4, p107/p130-binding | transcription |
| *EN2* | engrailed homeobox 2 | transcription |
| *IRF5* | interferon regulatory factor 5 | transcription |
| *MCM3* | minichromosome maintenance complex component 3 | transcription |
| *NRIP1* | nuclear receptor interacting protein 1 | transcription |
| *PELP1* | proline, glutamate and leucine rich protein 1 | transcription |
| *PWP1* | PWP1 homolog (S. cerevisiae) | transcription |
| *RNH1* | ribonuclease/angiogenin inhibitor 1 | transcription |
| *SREBF1* | sterol regulatory element binding transcription factor 1 | transcription |
| *TCEAL8* | transcription elongation factor A (SII)-like 8 | transcription |
| *TWIST1* | twist homolog 1 (Drosophila) | transcription |
| *VGLL3* | vestigial like 3 (Drosophila) | transcription |
| *CARHSP1* | calcium regulated heat stable protein 1, 24kDa | translation |
| *EIF2B1* | eukaryotic translation initiation factor 2B, subunit 1 alpha, 26kDa | translation |
| *EIF4A1* | eukaryotic translation initiation factor 4A1 | translation |
| *SIRT1* | sirtuin (silent mating type information regulation 2 homolog) 1 (S. cerevisiae) | histone deacetylase |
| *SRP9* | signal recognition particle 9kDa | translation |
| *TDRD7* | tudor domain containing 7 | translation |
| **Transport** | | |
| *ARF3* | ADP-ribosylation factor 3 | protein trafficking |
| *EXOC1* | exocyst complex component 1 | exocytosis |
| *LAPTM5* | lysosomal protein transmembrane 5 | lysosome transport |
| *NUP62* | nucleoporin 62kDa | mRNA transport |
| *CD52* | CD52 molecule | carbohydrates transport |
| *POP4* | processing of precursor 4, ribonuclease P/MRP subunit (S. cerevisiae) | RNA transport |
| *SLC19A2* | solute carrier family 19 (thiamine transporter), member 2 | thiamine transport |
| *SLC35C2* | solute carrier family 35, member C2 | response to hypoxia |
| *SLC4A4* | solute carrier family 4, sodium bicarbonate cotransporter, member 4 | Na-HCO3 transport |
| *TXNDC5* | thioredoxin domain containing 5 (endoplasmic reticulum) | vesicule-mediated transport |
| **Miscellaneous** | | |
| *BTBD7* | BTB (POZ) domain containing 7 | unknown |
| *CIDECP* | cell death-inducing DFFA-like effector c pseudogene | unknown |
| *CYYR1* | cysteine/tyrosine-rich 1 | unknown |
| *DNAJC13* | DnaJ (Hsp40) homolog, subfamily C, member 13 | unknown |
| *NOMO1* | NODAL modulator 1 | unknown |
| *SH3BGRL* | SH3 domain binding glutamic acid-rich protein like | unknown |
| *TMEM135* | transmembrane protein 135 | unknown |

The 221 transcripts were selected from previous published and unpublished DNA microarray analyses of human subcutaneous adipose tissue biopsies performed on limited number of individuals as described in [[1](#_ENREF_1)]. These genes encode proteins involved in various pathways including metabolism (43% of the transcripts), immune response (20%), cell differentiation and proliferation (9%), cell and tissue structure (7%), transport (5%), signal transduction (3%) and response to stress (1.3%). The list of genes extracted from the previous microarray data analysis includes 89 genes previously shown as markers of subcutaneous adipose tissue from obese insulin resistant individuals with metabolic syndrome [[2](#_ENREF_2)], 44 genes described as markers of subcutaneous adipose tissue from lean individuals [[2](#_ENREF_2)], 39 markers of weight changes after caloric restriction [[3](#_ENREF_3)], 40 genes selected from previous caloric restriction induced weight loss studies [[4](#_ENREF_4),[5](#_ENREF_5)], and 18 unpublished predictors of weight change to distinguish between those individuals that will regain weight after LCD from those that will succeed weight maintaining (based on the adipose tissue transcriptome at baseline, or after the caloric restriction phase).

1. Viguerie N, Montastier E, Maoret JJ, Roussel B, Combes M, et al. (2012) Determinants of human adipose tissue gene expression: impact of diet, sex, metabolic status, and cis genetic regulation. PLoS Genet 8: e1002959.

2. Klimcakova E, Roussel B, Marquez-Quinones A, Kovacova Z, Kovacikova M, et al. (2010) Worsening of obesity and metabolic status yields similar molecular adaptations in human subcutaneous and visceral adipose tissue: decreased metabolism and increased immune response. J Clin Endocrinol Metab 96: E73-82.

3. Marquez-Quinones A, Mutch DM, Debard C, Wang P, Combes M, et al. (2010) Adipose tissue transcriptome reflects variations between subjects with continued weight loss and subjects regaining weight 6 mo after caloric restriction independent of energy intake. Am J Clin Nutr 92: 975-984.

4. Capel F, Viguerie N, Vega N, Dejean S, Arner P, et al. (2008) Contribution of energy restriction and macronutrient composition to changes in adipose tissue gene expression during dietary weight-loss programs in obese women. J Clin Endocrinol Metab 93: 4315-4322.

5. Capel F, Klimcakova E, Viguerie N, Roussel B, Vitkova M, et al. (2009) Macrophages and adipocytes in human obesity: adipose tissue gene expression and insulin sensitivity during calorie restriction and weight stabilization. Diabetes 58: 1558-1567.
