# Supplementary material for: System Model Network for Adipose Tissue Signatures Related to Weight Changes in Response to Calorie Restriction and Subsequent Weight Maintenance
Source: PLoS Comput Biol. 2015 Jan 15;11(1):e1004047. doi: 10.1371/journal.pcbi.1004047 (PMC4295881; doi:10.1371/journal.pcbi.1004047)
Supplement: S3 Table — (DOCX) [file pcbi.1004047.s005.docx]

**Table S3.  Anthropometric and clinical characteristics at the end of the weight maintenance phase according to weight control group and by randomization arm.**

|  | WR (n=51) | | | | | WS (n=39) | | | | | WL (n=45) | | | | |
| --- | --- | --- | --- | --- | --- | --- | --- | --- | --- | --- | --- | --- | --- | --- | --- |
|  | LP-LGI | LP-HGI | HP-LGI | HP-HGI | CTRL | LP-LGI | LP-HGI | HP-LGI | HP-HGI | CTRL | LP-LGI | LP-HGI | HP-LGI | HP-HGI | CTRL |
| Age (y) | 40.2 ± 1.4 | 43.9 ± 1.8 | 42.5 ± 2.4 | 42.7 ± 2.4 | 44.2 ± 1.6 | 43.6 ± 1.7 | 36.1 ± 2.3 | 40.9 ± 3.8 | 46.4 ± 2.7 | 40.8 ± 1.7 | 43.9 ± 1.4 | 44.9 ± 3.9 | 44.5 ± 1.8 | 39.5 ± 1.9 | 44.6 ± 2.5 |
| Weight (kg) | 82.9 ± 6.8 | 86.9 ± 3.5 | 90.2 ± 5.2 | 88.8 ± 7.0 | 88.4 ± 2.9 | 79.1 ± 2.0 | 79.8 ± 2.2 | 77.8 ± 4.6 | 77.2 ± 4.0 | 86.5 ± 3.3 | 81.6 ± 4.9 | 77.2 ± 4.8 | 79.5 ± 4.5 | 87.9 ± 7.3 | 78.3 ± 4.6 |
| BMI (kg/m²) | 30.7 ± 2.4 | 32.4 ± 1.2 | 32.5 ± 1.6 | 31.8 ± 2.1 | 32.5 ± 1.0 | 29.2 ± 0.7 | 29.5 ± 1.2 | 30.3 ± 1.5 | 28.3 ± 1.7 | 31.1 ± 1.2 | 29.1 ± 1.2 | 27.8 ± 1.6 | 28.9 ± 1.2 | 32.0 ± 1.2 | 28.2 ± 1.5 |
| Fat Mass (%) | 41.2 ± 2.6 | 41.5 ± 1.7 | 42.2 ± 2.3 | 40.2 ± 3.1 | 41.7 ± 1.4 | 37.5 ± 0.9 | 39.7 ± 1.4 | 39.1 ± 1.5 | 37.1 ± 2.2 | 35.7 ± 2.5 | 34.9 ± 2.6 | 34.2 ± 3.8 | 37.9 ± 1.6 | 38.8 ± 2.4 | 35.5 ± 1.4 |
| Waist (cm) | 99 ± 6 | 98 ± 3 | 97 ± 3 | 94 ± 6 | 102 ± 2 | 92 ± 2 | 93 ± 3 | 92 ± 4 | 92 ± 5 | 91 ± 3 | 88 ± 3 | 91 ± 4 | 90 ± 4 | 97 ± 6 | 86 ± 4 |
| SBP (mmHg) | 119 ± 8 | 123 ± 3 | 116 ±3 | 113 ± 6 | 120 ± 4 | 123 ± 3 | 117 ± 8 | 117 ± 6 | 125 ± 6 | 117 ± 4 | 122 ± 4 | 116 ± 6 | 122 ± 2 | 121 ± 5 | 121 ± 4 |
| DBP (mmHg) | 74 ± 7 | 76 ± 2 | 70 ± 3 | 73 ± 3 | 74 ± 4 | 75 ± 2 | 72 ± 6 | 74 ± 7 | 74 ± 4 | 72 ± 2 | 75 ± 2 | 79 ± 8 | 72 ± 2 | 75 ± 3 | 75 ± 2 |
| Total Cholesterol (mmol/l) | 4.5 ± 0.2 | 5.1 ± 0.2 | 4.7 ± 0.2 | 4.8 ± 0.3 | 5.2 ± 0.2 | 5.3 ± 0.2 | 4.4 ± 0.1 | 5.4 ± 0.3 | 5.0 ± 0.2 | 4.2 ± 0.2 | 4.8 ± 0.3 | 5.0 ± 0.4 | 4.9 ± 0.3 | 4.7 ± 0.3 | 4.6 ± 0.3 |
| Triglycerides (mmol/l) | 1.3 ± 0.2 | 1.0 ± 0.1 | 1.3 ± 0.2 | 1.3 ± 0.3 | 1.2 ± 0.1 | 0.9 ± 0.1 | 0.8 ± 0.1 | 1.0 ± 0.1 | 0.9 ± 0.2 | 0.9 ± 0.1 | 1.1 ± 0.1 | 1.1 ± 0.1 | 1.1 ± 0.2 | 1.0 ± 0.2 | 0.9 ± 0.1 |
| HDL (mmol/l) | 1.5 ± 0.1 | 1.5 ± 0.05 | 1.4 ± 0.1 | 1.3 ± 0.2 | 1.5± 0.1 | 1.6 ± 0.1 | 1.4 ± 0.2 | 1.3 ± 0.2 | 1.6 ± 0.2 | 1.4 ± 0.1 | 1.5 ± 0.1 | 1.8 ± 0.1 | 1.4 ± 0.1 | 1.3 ± 0.1 | 1.5 ± 0.1 |
| LDL (mmol/l) | 2.5 ± 0.2 | 3.2 ± 0.2 | 2.7 ± 0.2 | 2.9 ± 0.3 | 3.2 ± 0.2 | 3.3 ± 0.2 | 2.3 ± 0.2 | 3.6 ± 0.2 | 3.0 ± 0.2 | 2.4 ± 0.2 | 2.8 ± 0.3 | 2.7 ± 0.4 | 3.0 ± 0.2 | 3.0 ± 0.3 | 2.8 ± 0.3 |
| Fasting glucose (mmol/l) | 4.8 ± 0.1 | 4.9 ± 0.1 | 4.8 ± 0.2 | 4.9 ± 0.1 | 5.1 ± 0.2 | 5.0 ± 0.2 | 4.7 ± 0.2 | 4.8 ± 0.1 | 5.1 ± 0.2 | 4.9 ± 0.2 | 4.9 ± 0.1 | 4.8 ± 0.2 | 5.1 ± 0.3 | 4.7 ± 0.2 | 4.6 ± 0.2 |
| Fasting insulin (µIU/ml) | 9.6 ± 2.4 | 8.2 ± 1.1 | 7.8 ± 1.1 | 9.3 ± 1.7 | 8.7 ± 1.3 | 7.5 ± 1.2 | 7.8 ± 3.6 | 6.5 ± 1.5 | 6.1 ± 0.8 | 6.5 ± 1.0 | 8.6 ± 2.4 | 6.9 ± 1.0 | 9.4 ± 1.8 | 9.7 ± 2.3 | 6.7 ± 1.9 |
| HOMA-IR | 2.0 ± 0.5 | 1.6 ± 0.2 | 1.6 ± 0.3 | 2.0 ± 0.5 | 1.9 ± 0.3 | 1.7 ± 0.3 | 1.7 ± 0.9 | 1.3 ± 0.3 | 1.3 ± 0.2 | 1.3 ± 0.2 | 1.9 ± 0.6 | 1.6 ± 0.3 | 2.2 ± 0.6 | 1.9 ± 0.5 | 1.4 ± 0.4 |
| Fructosamine (µmol/l) | 211 ± 9 | 212 ± 5 | 212 ± 4 | 212 ± 6 | 224 ± 5 | 219 ± 5 | 205 ± 9 | 234 ± 4 | 216 ± 8 | 221 ± 10 | 223 ± 7 | 221 ± 8 | 215 ± 6 | 207 ± 7 | 222 ± 5 |
| Adiponectin (µg/ml) | 12.0 ± 2.1 | 13.1 ± 1.4 | 11.7 ± 1.4 | 10.5 ± 1.3 | 10.3 ± 1.0 | 9.1 ± 1.0 | 11.4 ± 3.1 | 8.3 ± 1.1 | 12.8 ± 2.2 | 11.6 ± 1.6 | 14.1 ± 1.5 | 14.1 ± 2.3 | 14.2 ± 2.8 | 11.2 ± 0.7 | 13.7 ± 0.9 |
| CRP (mg/l) | 3.8 ± 1.4 | 2.3 ± 0.6 | 3.0 ± 1.2 | 3.9 ± 1.1 | 2.7 ± 0.7 | 2.2 ± 0.9 | 3.0 ± 1.2 | 3.4 ± 1.3 | 1.8 ± 0.6 | 1.2 ± 0.3 | 2.9 ± 1.0 | 2.2 ± 1.5 | 5.3 ± 1.0 | 4.2 ± 1.6 | 1.2 ± 0.3 |

Anthropometric, clinical and plasma parameters were determined after 6 months of weight maintenance diet (WMD) according to weight control groups (WR, weight regain group; WS, weight stable group; WL, weight loss group) and to type of diet (LP-LGI, low protein-low glycemic index; LP-HGI, low protein-high glycemic index; HP-LGI, high protein-low glycemic index; HP-HGI, high protein-high glycemic index; CTRL, control diet). BMI, body mass index; CRP, C reactive protein; DBP, diastolic blood pressure; HDL, high density lipoprotein; HOMA-IR, homeostatic model assessment of insulin resistance; LDL, low density lipoprotein; SBP, systolic blood pressure.

Variables are shown as means ± SEM. Differences were tested by two-way ANOVA with Bonferroni post hoc test. No significant difference was found.
