# Supplementary material for: System Model Network for Adipose Tissue Signatures Related to Weight Changes in Response to Calorie Restriction and Subsequent Weight Maintenance
Source: PLoS Comput Biol. 2015 Jan 15;11(1):e1004047. doi: 10.1371/journal.pcbi.1004047 (PMC4295881; doi:10.1371/journal.pcbi.1004047)
Supplement: S4 Table — (DOCX) [file pcbi.1004047.s006.docx]

**Table S4**. Adipose tissue fatty acid content according to weight control groups during dietary intervention.

|  | | |  | WR (n=51) | | | WS (n=39) | | | | WL (n=45) | | | |
| --- | --- | --- | --- | --- | --- | --- | --- | --- | --- | --- | --- | --- | --- | --- |
|  | | |  | BAS | LCD | WMD | BAS | | LCD | WMD | BAS | LCD | WMD | |
| **SFAs** | | | |  | | |  | | | |  | | | |
|  | 12:0 | | | 0.32±0.02 | 0.25±0.02 | 0.34±0.02*^2,3^* | 0.35±0.03 | | 0.30±0.03*^3^* | 0.31±0.03 | 0.29±0.02 | 0.22±0.02*^1^* | | 0.25±0.02 |
|  | 14:0 | | | 2.67±0.10*^3^* | 2.45±0.08*^3^* | 2.61±0.08*^3^* | 2.77±0.10 *^3^* | | 2.53±0.11*^1,3^* | 2.53±0.12*^1,3^* | 2.33±0.11 | 2.12±0.08*^1^* | | 2.12±0.10*^1^* |
|  | 16:0 | | | 23.40±0.48*^3^* | 23.20±0.37*^3^* | 22.96±0.27*^3^* | 23.54±0.36*^3^* | | 22.80±0.36*^1,3^* | 22.77±0.37*^1,3^* | 21.93±0.31 | 21.41±0.30 | | 21.25±0.30 |
|  | 18:0 | | | 3.74±0.11 | 3.77±0.10 | 3.80±0.11 | 3.79±0.11 | | 3.94±0.12 | 3.87±0.13 | 3.71±0.14 | 3.77±0.13 | | 3.94±0.13 |
|  | 20:0 | | | 0.39±0.05 | 0.33±0.04 | 0.34±0.04 | 0.35±0.06 | | 0.25±0.04 | 0.29±0.05 | 0.38±0.05 | 0.34±0.04 | | 0.35±0.04 |
|  | Total SFAs | | | 30.54±0.61*^3^* | 30.00±0.46*^3^* | 30.06±0.41*^3^* | 30.79±0.49*^3^* | | 29.87±0.51*^1,3^* | 29.80±0.53*^1,3^* | 28.63±0.49 | 27.86±0.46*^1^* | | 27.91±0.46 |
| **MUFAs** | | | |  | | |  | | | |  | | | |
|  | 14:1(cis-9) | | | 0.29±0.01*^3^* | 0.26±0.01*^3^* | 0.29±0.01*^2,3^* | 0.30±0.02*^3^* | | 0.26±0.02*^1,3^* | 0.26±0.02*^1,3^* | 0.24±0.02 | 0.20±0.01*^1^* | | 0.20±0.01*^1^* |
|  | 16:1(cis-9) | | | 4.82±0.15*^3^* | 4.66±0.18*^3^* | 4.69±0.16*^3^* | 4.72±0.22 | | 4.30±0.21*^1,3^* | 4.45±0.21*^3^* | 4.20±0.18 | 3.88±0.16*^1^* | | 3.71±0.15*^1^* |
|  | 16:1(trans-9) | | | 0.033±0.002 | 0.033±0.002*^3^* | 0.029±0.002 | 0.031±0.002 | | 0.029±0.003 | 0.028±0.002 | 0.029±0.002 | 0.025±0.002*^1^* | | 0.023±0.002*^1^* |
|  | 16:1(cis-7) | | | 0.66±0.02 | 0.69±0.01 | 0.67±0.02 | 0.64±0.01 | | 0.67±0.01*^1^* | 0.67±0.02 | 0.63±0.01 | 0.66±0.01*^1^* | | 0.65±0.01 |
|  | 18:1(cis-11) | | | 1.94±0.05*^3^* | 1.94±0.05*^3^* | 1.91±0.05*^3^* | 1.90±0.06 | | 1.84±0.08 | 1.89±0.07*^3^* | 1.74±0.05 | 1.74±0.04 | | 1.68±0.05 |
|  | 18:1(cis-9) | | | 45.47±0.46 | 46.51±0.47*^1,3^* | 45.43±0.43*^2^* | 44.77±0.54 | | 45.87±0.56*^1^* | 45.60±0.55*^1^* | 43.71±0.65 | 44.46±0.66*^1^* | | 44.80±0.70*^1^* |
|  | 18:1(trans-9) +  18:1(trans-11) | | | 1.08±0.06 | 1.11±0.04 | 1.06±0.04 | 1.10±0.05 | | 1.08±0.05 | 1.03±0.04 | 0.96±0.03 | 1.05±0.04 | | 1.06± 0.06 |
|  | 20:1(cis-11) | | | 0.65±0.02 | 0.69±0.02*^1^* | 0.66±0.02 | 0.12±0.02 | | 0.13±0.02*^1^* | 0.12±0.02*^1^* | 0.61±0.02 | 0.66±0.02*^1^* | | 0.69± 0.02*^1^* |
|  | Total MUFAs | | | 54.94±0.47*^3^* | 55.89±0.52*^1,3^* | 54.73±0.49*^1^* | 54.06±0.61 | | 54.75±0.67*^1^* | 54.63±0.67*^1^* | 52.12±0.74 | 52.68±0.79 | | 52.81±0.79 |
| **PUFAs** | | | |  | | |  | | | |  | | | |
|  | | 18:2(cis-9,12) | | 12.52±0.57*^3^* | 12.14±0.57*^3^* | 13.09±0.48*^3^* | 13.11±0.86*^3^* | 13.19±0.92*^3^* | | 13.34±0.83*^3^* | 17.33±0.86 | 17.38±0.85 | | 17.24±0.79 |
|  | | 18:2(trans-9,12) | | 0.013±0.001 | 0.010±0.001 | 0.011±0.001 | 0.015±0.002 | 0.010±0.001 | | 0.011±0.001 | 0.010±0.001 | 0.009±0.001 | | 0.010±0.001 |
|  | | 18:2(trans-9,cis-12) | | 0.102±0.010*^3^* | 0.079±0.009 | 0.095±0.012 | 0.102±0.010*^3^* | 0.101±0.012*^3^* | | 0.095±0.013 | 0.059±0.010 | 0.057±0.010 | | 0.070±0.014 |
|  | | 18:2(cis-9,trans-12) | | 0.045±0.004*^3^* | 0.035±0.004 | 0.034±0.004*^1,3^* | 0.049±0.005*^3^* | 0.040±0.005*^3^* | | 0.037±0.004*^1,3^* | 0.027±0.004 | 0.023 ± 0.003 | | 0.021±0.003 |
|  | | 18:3(cis-6,9,12) | | 0.052±0.005 | 0.043±0.002*^3^* | 0.050±0.003 | 0.025±0.004 | 0.023±0.004*^3^* | | 0.025±0.004 | 0.055±0.003 | 0.057±0.003 | | 0.056±0.002 |
|  | | 18:3(cis-9,12,15) | | 0.42±0.05 | 0.37±0.04 | 0.43±0.04*^3^* | 0.44±0.05 | 0.39±0.04*^1, 3^* | | 0.41±0.04*^1^* | 0.28±0.04 | 0.26±0.04 | | 0.25±0.04 |
|  | | 20:2(cis-11,14) | | 0.20±0.01*^3^* | 0.20±0.01*^3^* | 0.21±0.01*^3^* | 0.08±0.01 | 0.10±0.02*^1,3^* | | 0.09±0.02*^1,3^* | 0.26±0.01 | 0.29±0.01*^1^* | | 0.29±0.01*^1^* |
|  | | 20:3(cis-5,8,11) | | 0.024±0.002 | 0.025±0.002 | 0.026±0.002 | 0.012±0.002 | 0.019±0.003 | | 0.020±0.003 | 0.020±0.001 | 0.021±0.002 | | 0.018±0.002 |
|  | | 20:3(cis-8,11,14) | | 0.22±0.01*^3^* | 0.25±0.01*^3^* | 0.25±0.01*^3^* | 0.09±0.01 | 0.10±0.02*^1^* | | 0.10±0.02*^1,4^* | 0.30±0.02 | 0.34±0.02*^1^* | | 0.33±0.02*^1^* |
|  | | 20:4(cis-5,8,11,14) | | 0.40±0.02 | 0.42±0.02*^3^* | 0.45±0.02 | 0.11±0.02 | 0.17±0.03*^1^* | | 0.14±0.02*^1^* | 0.44±0.02 | 0.50±0.02*^1^* | | 0.47±0.02 |
|  | | 20:5(cis-5,8,11,14,17) | | 0.060±0.005 | 0.053±0.004 | 0.066±0.005 | 0.035±0.006 | 0.019±0.003 | | 0.032±0.005 | 0.046±0.004 | 0.044±0.004 | | 0.050±0.006 |
|  | | 22:4(cis-7,10,13,16) | | 0.150±0.009 | 0.150±0.007*^3^* | 0.157±0.007*^3^* | 0.060±0.010 | 0.107±0.017 | | 0.062±0.010*^1^* | 0.176±0.012 | 0.197±0.012*^1^* | | 0.192±0.010 |
|  | | 22:5(cis-4,7,10,13,16) | | 0.041±0.008 | 0.036±0.002 | 0.031±0.003 | 0.055±0.004 | 0.057±0.003 | | 0.050±0.003 | 0.028±0.002 | 0.028±0.002 | | 0.028±0.002 |
|  | | 22:5(cis-7,10,13,16,19) | | 0.154±0.008 | 0.172±0.011 | 0.171±0.010 | 0.055±0.009 | 0.057±0.009*^1,3^* | | 0.050±0.008*^1^* | 0.132±0.009 | 0.158±0.008*^1^* | | 0.147±0.008 |
|  | | 22:6(cis-4,7,10,13,16,19) | | 0.120±0.007 | 0.126±0.011 | 0.139±0.009*^3^* | 0.027±0.009 | 0.020±0.019 | | 0.016±0.015*^3^* | 0.096±0.010 | 0.099±0.006 | | 0.102±0.007 |
|  | | Total PUFAs | | 14.53±0.58*^3^* | 14.11±0.59*^3^* | 15.21±0.48*^3^* | 15.15±0.87*^3^* | 15.38±0.94*^3^* | | 15.58±0.84*^3^* | 19.26±0.87 | 19.45±0.86 | | 19.28±0.79 |

Individual fatty acids (molar %) from adipose tissue fat cake were determined using gaz chromatography and calculated according to groups at baseline (BAS), after 8 weeks low calorie diet (LCD), and after 6 months of weight maintenance diet (WMD).

Variables are shown as means ± SEM.

~~P-value was calculated by repeated measures ANOVA with Bonferroni post hoc test.~~

The effect of time was analyzed by repeated measures ANOVA with Bonferroni post hoc test. Data are presented as false discovery rate adjusted p-value (q-value) using Benjamini-Hochberg procedure.

*^1^* q < 0.05, data significantly different from BAS

*^2^* q < 0.05, data significantly different from LCD

Between group difference was analyzed by one-way ANOVA with Bonferroni post hoc test. Data are presented as false discovery rate adjusted p-value (q-value) using Benjamini-Hochberg procedure.

*^3^* q < 0.05, data significantly different from WL group

*^4^* q < 0.05, data significantly different from WR group
