# Supplementary figures and images for: System Model Network for Adipose Tissue Signatures Related to Weight Changes in Response to Calorie Restriction and Subsequent Weight Maintenance
Source: PLoS Comput Biol. 2015 Jan 15;11(1):e1004047. doi: 10.1371/journal.pcbi.1004047 (PMC4295881; doi:10.1371/journal.pcbi.1004047)

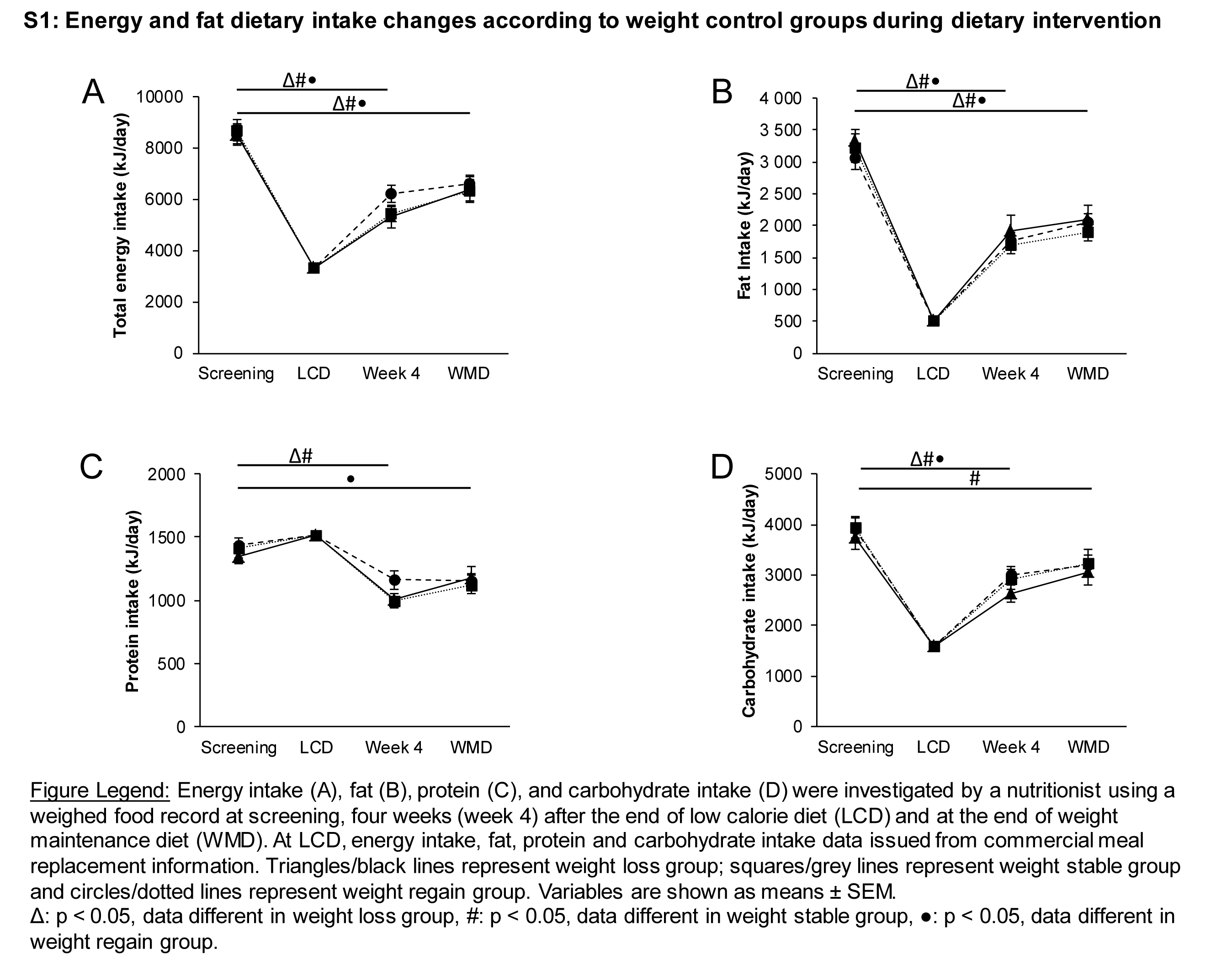

Supplement: S1 Fig — Energy intake (A), fat (B), protein (C), and carbohydrate intake (D) were investigated by a nutritionist using a weighed food record at screening, four weeks (week 4) after the end of low calorie diet (LCD) and at the end of weight maintenance diet (WMD). At LCD, energy intake, fat, protein and carbohydrate intake data issued from commercial meal replacement information. Triangles/black lines represent weight loss group; squares/grey lines represent weight stable group and circles/dotted lines represent weight regain group. Variables are shown as means ± SEM. Δ: p < 0.05, data different in weight loss group, #: p < 0.05, data different in weight stable group, ●: p < 0.05, data different in weight regain group. (TIF) [file pcbi.1004047.s007.tif]

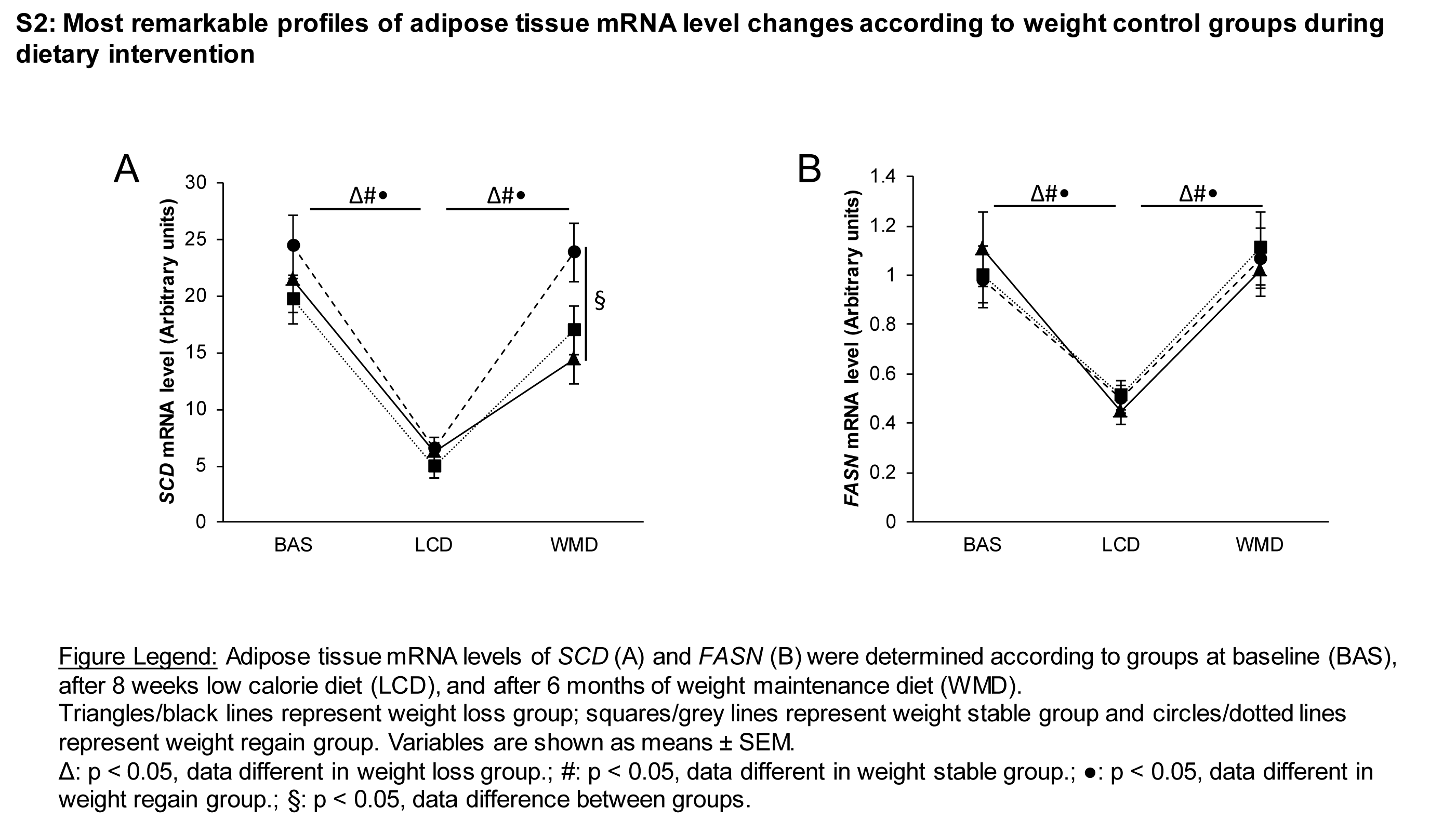

Supplement: S2 Fig — Adipose tissue mRNA levels of SCD (A) and FASN (B) were determined according to groups at baseline (BAS), after 8 weeks low calorie diet (LCD), and after 6 months of weight maintenance diet (WMD). Triangles/black lines represent weight loss group; squares/grey lines represent weight stable group and circles/dotted lines represent weight regain group. Variables are shown as means ± SEM. Δ: p < 0.05, data different in weight loss group.; #: p < 0.05, data different in weight stable group.; ●: p < 0.05, data different in weight regain group.; §: p < 0.05, data difference between groups. (TIF) [file pcbi.1004047.s008.tif]

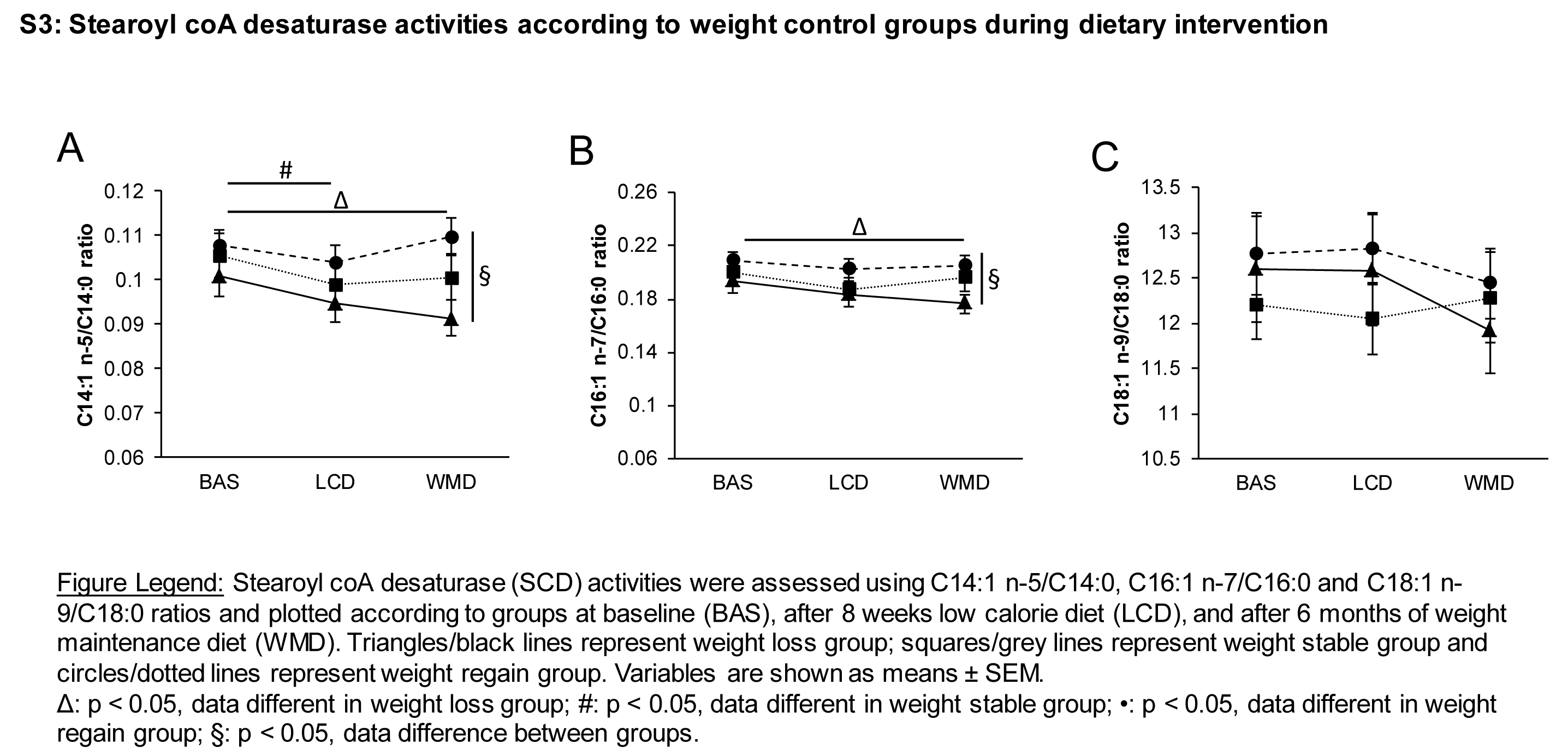

Supplement: S3 Fig — Stearoyl coA desaturase (SCD) activities were assessed using C14:1 n-5/C14:0, C16:1 n-7/C16:0 and C18:1 n-9/C18:0 ratios and plotted according to groups at baseline (BAS), after 8 weeks low calorie diet (LCD), and after 6 months of weight maintenance diet (WMD). Triangles/black lines represent weight loss group; squares/grey lines represent weight stable group and circles/dotted lines represent weight regain group. Variables are shown as means ± SEM. Δ: p < 0.05, data different in weight loss group; #: p < 0.05, data different in weight stable group; •: p < 0.05, data different in weight regain group; §: p < 0.05, data difference between groups. (TIF) [file pcbi.1004047.s009.tif]
